# Association Between In-Utero Exposure to Antibiotics and Offspring’s Hearing Loss: A Systematic Review and Meta-Analysis

**DOI:** 10.3390/children12030356

**Published:** 2025-03-13

**Authors:** Jing Wang, Nur Farah Addina Lee Binte Zailan, Yichao Wang, Samuel Lake, Yanhong Jessika Hu

**Affiliations:** 1 Murdoch Children’s Research Institute, Royal Children’s Hospital, 50 Flemington Road, Parkville 3052, VIC, Australia; jing.wang@mcri.edu.au (J.W.); yichao.wang1@deakin.edu.au (Y.W.); slake1@student.unimelb.edu.au (S.L.); 2 Department of Pediatrics, The University of Melbourne, Parkville 3052 VIC, Australia; 3 Department of Pediatrics, Yong Loo Lin School of Medicine, National University of Singapore, Singapore 119077, Singapore; addina@nus.edu.sg; 4 Centre for Social and Early Emotional Development, School of Psychology, Deakin University, Geelong 3220, VIC, Australia

**Keywords:** antibiotics, antimicrobials, antenatal exposure, prenatal exposure, maternal–fetal transmission, pregnancy, children, birth outcomes, hearing loss

## Abstract

**Objectives:** Antibiotic exposure during pregnancy is common, accounting for over 80% of all medications prescribed. Antibiotics in pregnancy are linked to increased childhood disease risk, through direct toxicity or potentially microbiome dysbiosis. This systematic review investigated the relationship between in-utero exposure to antibiotics and childhood hearing loss. **Methods:** We searched Ovid Medline, Embase, and PubMed for studies examining antibiotic exposure during pregnancy and its associations with hearing loss in offspring. Studies with children whose mothers had data on antibiotic exposure during pregnancy were selected. The meta-analysis calculated (1) pooled prevalence of childhood hearing loss and (2) pooled odds ratios (ORs) for associations between in-utero exposure to antibiotics and childhood hearing loss. **Results:** Of 1244 studies identified, 18 met the inclusion criteria. Among 161,053 children exposed in-utero to antibiotics, 4368 developed hearing loss. The pooled prevalence of childhood hearing loss was 0.9% (95% CI 0.0–2.8%, I^2^ = 99.6%). In-utero exposure to antibiotics was associated with an increased risk of childhood hearing loss (pooled OR 1.2, 95% CI 1.1 to 1.3, I^2^ = 15.4%). Aminoglycoside exposure during pregnancy was associated with a higher risk of hearing loss (pooled OR 1.2, 95% CI 1.1 to 1.3, I^2^ = 38.4%), while exposure to other antibiotic classes showed no association. **Conclusions:** The prevalence of childhood hearing loss among those exposed to antibiotics during pregnancy is high. Although the overall risk appears modest, aminoglycosides are linked to a significantly higher risk, suggesting maternal aminoglycoside exposure may indicate a risk for child hearing loss. Further research is needed to clarify causal pathways and long-term effects of in-utero exposure to antibiotics.

## 1. Introduction

Hearing loss is one of the most prevalent childhood diseases worldwide, with disabling hearing loss—defined as hearing level exceeding 35 decibels—affecting 34 million children in 2021 [1]. The economic burden of hearing loss is significant. According to the World Health Organization, moderate and severe hearing loss in children aged 0 to 14 incurs costs ranging from 24 to 45 billion international dollars annually [2]. The consequences of childhood hearing loss extend far beyond financial burdens, as it can lead to significant challenges such as learning difficulties, impaired communication skills, and adverse neurodevelopmental outcomes [3,4]. Notably, 60% of childhood hearing loss is preventable [5]. Therefore, identifying the causal and modifiable risk factors of hearing loss is essential.

One potential modifiable cause of hearing loss is in-utero exposure to antibiotics. In-utero exposure to antibiotics accounts for about 80% of drugs administered to pregnant women [6]. Studies suggest that antibiotic exposure during pregnancy may elevate the risk of childhood hearing loss from a direct toxic effect on the cells of the ear [7,8], which disrupts the balance of microbial communities essential for health and disruption of the “gut–brain axis” [9], a critical pathway for neurodevelopment and sensory processing. Additionally, certain antibiotics have direct teratogenic effects that may adversely impact fetal ear development, potentially leading to structural anomalies. A study by Conway et al. [10] reported increased risks of congenital ear malformations following in-utero exposure to streptomycin. Previous population-based studies have shown that children exposed to antibiotics in-utero have up to a 20% higher risk of pediatric infections [11], including middle ear infections [12], which are linked to childhood hearing loss. These findings highlighted the needs for a careful evaluation of the risks and benefits of antibiotic use during pregnancy to minimize potential long-term auditory consequences for the offspring.

However, the association between antibiotic exposure during pregnancy and childhood hearing loss risk remains unclear. The association could be influenced by various factors including antibiotic class, dosage, the timing of administration during pregnancy, different hearing loss phenotypes (e.g., severity, laterality, and types) and genetic factors [13]. If in-utero exposure to antibiotics is found to increase the risk of hearing loss in offspring, it could promote better prescription management and safer antibiotic use among pregnant women, ultimately helping to reduce childhood hearing loss.

Therefore, this systematic review aimed to assess the current literature for evidence regarding the association between antibiotic exposure during pregnancy and childhood hearing loss.

## 2. Materials and Methods

This systematic review and meta-analysis was conducted according to the Preferred Reporting Items for Systematic Reviews and Meta-Analysis (PRISMA) statement [14].

### 2.1. Protocol and Registration

Methods for this systematic review and meta-analysis are detailed in the protocol registered with PROSPERO, the International Prospective Register of Systematic Reviews, on 8 June 2024, reference number CRD42024550079.

### 2.2. Search Strategy

We systematically searched for studies published between 1990 and 15 August 2024, in the databases of Ovid Medline, Embase, and PubMed. Medical Subject Headings and free text words were used to interrogate each database, including search terms for pregnant women and their offsprings (e.g., pregnancy, maternal, prenatal, newborns, babies, toddlers, children, adolescents, and teenagers), antibiotics (including broad classes and specific drugs, see Supplementary Table S1 for details), and hearing loss (e.g., hearing loss, hearing impairment, and deafness). The full search strategy for each database is provided in Supplementary Tables S2–S4.

### 2.3. Eligibility Criteria

Studies were considered eligible if they fulfilled all of the following criteria: (1) had pregnant women taking antibiotics during pregnancy, (2) recorded hearing loss outcome in offspring at birth or during childhood (0 to 21 years old); (3) study designs include case reports, case–control studies, cross-sectional studies, cohort studies, or randomized controlled trials; (4) reported the association between exposure and outcome or descriptive statistics of the number of children with or without hearing loss and the number of mothers who took or did not take antibiotics during pregnancy; and (5) had full text available in English.

We excluded studies if they met any of the following criteria (1) commentaries, letters, study protocols, treatment guidelines, conference reports, or reviews; (2) non-human studies (e.g., animals and tissue/cell lines); (3) antibiotic exposure not during pregnancy; or (4) reported hearing loss caused by risk factors other than antibiotic exposure (e.g., injury, infection, etc.).

### 2.4. Study Selection

Studies were uploaded to an online platform, Covidence, which automatically removed duplicates. Two independent reviewers (N.Z. and S.L.) screened the title and abstract, then the full text, of each study according to the eligibility criteria. We calculated an inter-rater reliability score from the screening results using Cohen’s Kappa for two independent reviewers in STATA SE 18. A Cohen’s Kappa value larger than 0.80 (or above 80%) was considered high agreement [15]. Any conflicts were discussed and resolved in consultation with a third reviewer (Y.J.H.).

### 2.5. Data Extraction

A data extraction form was used to extract study characteristics (authors, publication year, country, study design, sample size, and age of diagnosis of hearing loss in children), antibiotics exposure during pregnancy (e.g., name, dosage, and duration), hearing loss (e.g., testing frequencies, cut-off point, reported ear involved, types of hearing loss, and self-reported hearing loss), the number of reported hearing loss cases, reported associations between in-utero exposure to antibiotics and child hearing loss and confounding adjustment (if any). Data was extracted by N.Z. and independently verified by J.W and Y.J.H.

### 2.6. Risk of Bias Assessment Tools

Two reviewers (N.Z. and S.L) assessed each included study for methodological quality using three tools. Any discrepancy was resolved after discussion with Y.J.H. These included the Joanna Briggs Institute Critical Appraisal Checklist for Case Reports 2017 (JBICAC) [16], the Newcastle–Ottawa Scale for case–control and cohort studies [17], and the Cochrane Risk of Bias (RoB) tool for randomized controlled trials (RCTs) [18]. For cross-sectional analysis, we used a revised version of the Newcastle–Ottawa Scale. Details of each study’s risk of bias assessment are included in Supplementary Table S6.

### 2.7. Dealing with Missing Data

When information regarding the variables for analysis was missing from publications, the corresponding authors were emailed to request the data. If the authors did not reply after a second contact attempt, these publications were excluded from the relevant analyses.

### 2.8. Addressing Differences in Antibiotic Exposure Definitions

As studies varied in antibiotic exposure definitions, we divided them into subgroups to allow comparison and synthesis of results. Studies were mainly grouped according to antibiotic classes provided by the Therapeutic Goods Administration (TGA) of Australia [19] including aminoglycosides, beta-lactams, and macrolides. Antibiotics not classified by TGA were regarded as “Unspecified”, while studies examining multiple antibiotics collectively were assigned to the “Multiple Antibiotics” category. For the analysis, we combined the unspecified and multiple antibiotics groups under the category “Other antibiotics”.

### 2.9. Statistical Analysis

Prevalence of hearing loss in children with in-utero exposure to antibiotics:

We calculated the prevalence of hearing loss using the following formulae for each outcome reported in each study:

Prevalence of hearing loss = (Number of children with hearing loss)/(Number of children with in-utero exposure to antibiotics) × 100%

In studies reporting zero events, a continuity correction of 0.5 was added to both the numerators and denominators to facilitate statistical analysis [20,21]. The pooled prevalence of hearing loss was estimated by meta-analysis, accounting for anticipated heterogeneity across studies. To address this variability, we applied a random-effects model using the DerSimonian–Laird method to ensure robust and generalizable findings [22].

Associations between antibiotic use in pregnancy and child hearing loss: Meta-analysis and descriptive analysis were used to analyze the data. Odds ratios (ORs) were calculated from studies that reported the relevant descriptive data. A random-effects model using the Restricted Maximum Likelihood (REML) method was used to generate pooled effect sizes including 95% confidence intervals and *p*-values. The random-effects model [23] was used over other models as it takes variance between studies into consideration.

Heterogeneity between studies was measured using the I^2^ statistic. An I^2^ value of 75% and above is considered significant heterogeneity, 30% to 60% is regarded as modest heterogeneity, and 0% to 30% is non-significant [24]. We conducted subgroup analyses based on antibiotic classes. Publication bias was conducted by using eager’s test and trim-and-fill analysis [25]. All statistical analyses were performed using Stata SE 18 (StataCorp LLC, College Station, TX, USA).

## 3. Results

### 3.1. Search Results

**Figure 1:**
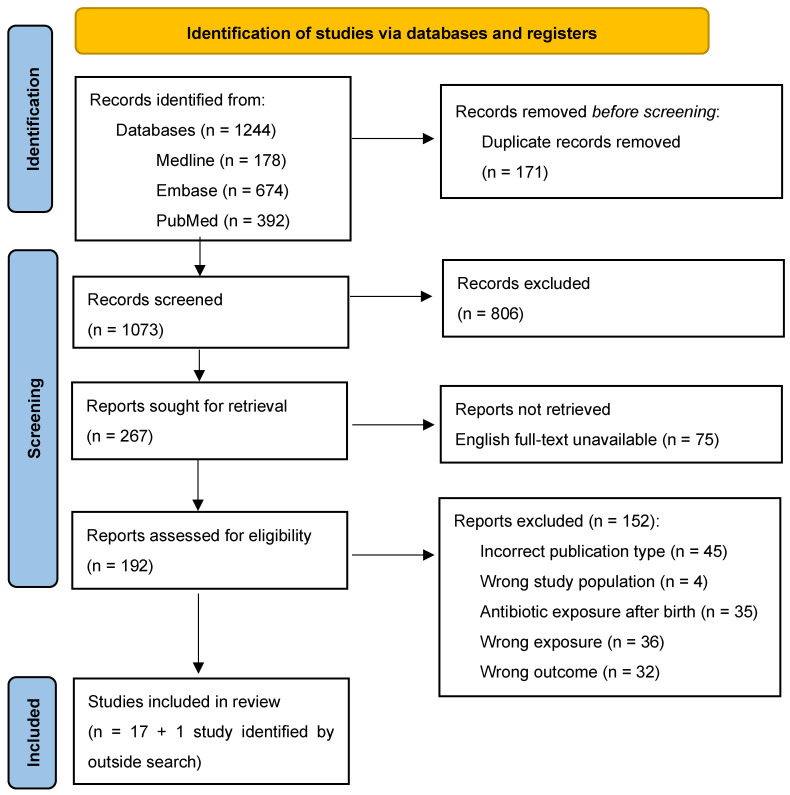
PRISMA flow diagram.

Figure 1 outlines the process of study selection through study identification, title/abstract screening, and full-text screening. From 1244 records identified by searching the three databases (Medline, Embase, and PubMed), 1073 papers underwent title/abstract screening after removing 171 duplicate records. A total of 267 passed title/abstract screening and underwent full-text screening, resulting in 17 studies eligible for inclusion. We further included one study from the grey literature.

### 3.2. Characteristics of Included Studies

Across the 18 included studies, participants were drawn from 13 countries. The studies comprised six cohort studies, five case–control studies, four cross-sectional studies, two case reports, and one RCT. Sample sizes ranged from 17 to 130,067 children in the cohort, case–control, and cross-sectional studies, one to two cases in the case reports, and 125 participants in the RCT. Among the antibiotic classes, aminoglycosides were the most commonly (n = 36,343) investigated, with four studies involving streptomycin exposure, ten including gentamicin, one on kanamycin, and one examining both gentamicin and kanamycin. In addition, one study assessed the effects of multiple antibiotics. Hearing loss was diagnosed at varying ages, with some studies focusing on newborns and others extending into childhood and adolescence. All included studies had only a single time-point of follow up (Table 1 and Supplementary Table S7). 

### 3.3. Prevalence of Hearing Loss in Children with In-Utero Exposure to Antibiotics 

**Figure 2:**
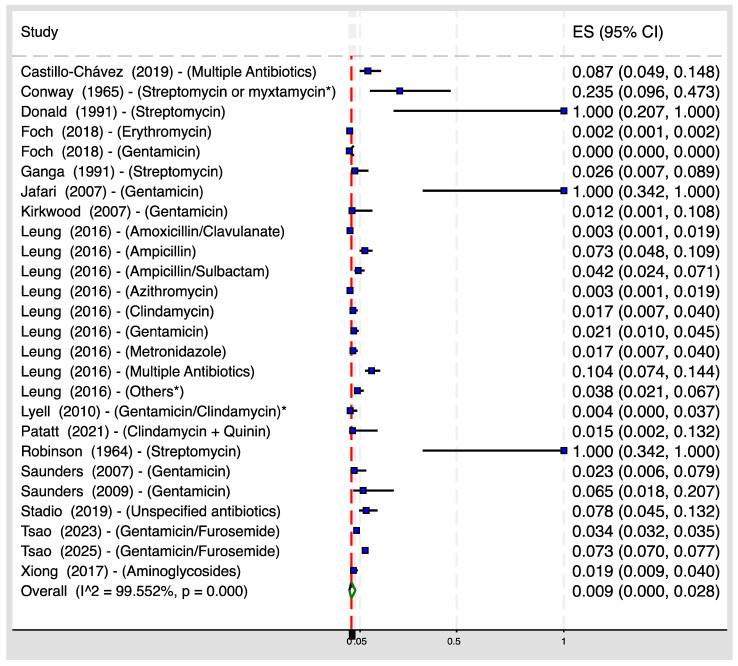
Pooled prevalence of childhood hearing loss from all included studies [10,26,27,28,29,30,31,32,33,34,35,36,37,38,39,40,42]. Note: ES: effect size; myxtamycin*: equal proportion of streptomycin and dihydrostreptomycin; Others*: *sulfamethoxazole, trimethoprim, cefotetan, ceftriaxone, cephalexin, cefazolin; Gentamicin/Clindamycin*: during labor.

Figure 2 shows that among 161,053 children who were exposed to various classes of antibiotics during their mothers’ pregnancy, 4368 of those developed hearing loss, resulting in a pooled hearing loss prevalence of 0.9% (95% CI 0.0–2.8%, I^2^ = 99.6%). When dividing the pooled prevalence by antibiotic class (Supplementary Figure S1), the pooled prevalences were: 0.1% (95% CI, 0.0–2.7%, I^2^ = 99.7%) for aminoglycosides, 3.0% (95% CI, 0.3–8.8%, I^2^ =92.1%) for beta-lactams, 0.1% (95% CI, 0.0–0.1%, I^2^ =0.0%) for macrolides, and 4.0% (95% CI, 1.9–7.7%, I^2^ = 84.6%) for other antibiotics.

### 3.4. Associations Between In-Utero Exposure to Antibiotics and Child Hearing Loss

**Figure 3:**
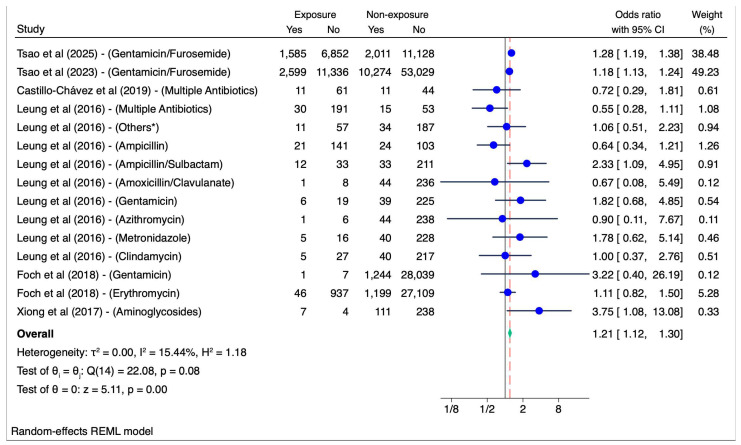
Overall association between antibiotics during pregnancy and offspring hearing loss [26,27,28,29,30,31]. Others*: *sulfamethoxazole, trimethoprim, cefotetan, ceftriaxone, cephalexin, cefazolin.

**Figure 4:**
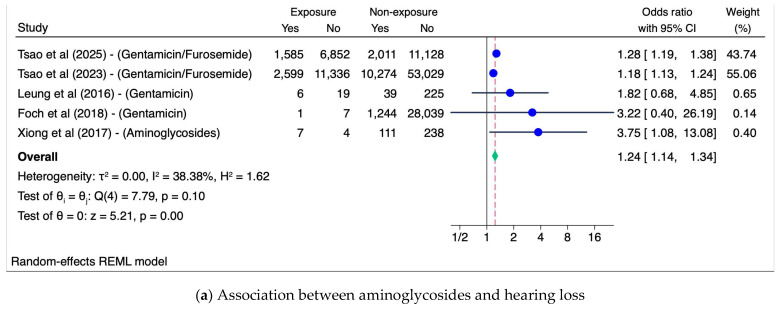

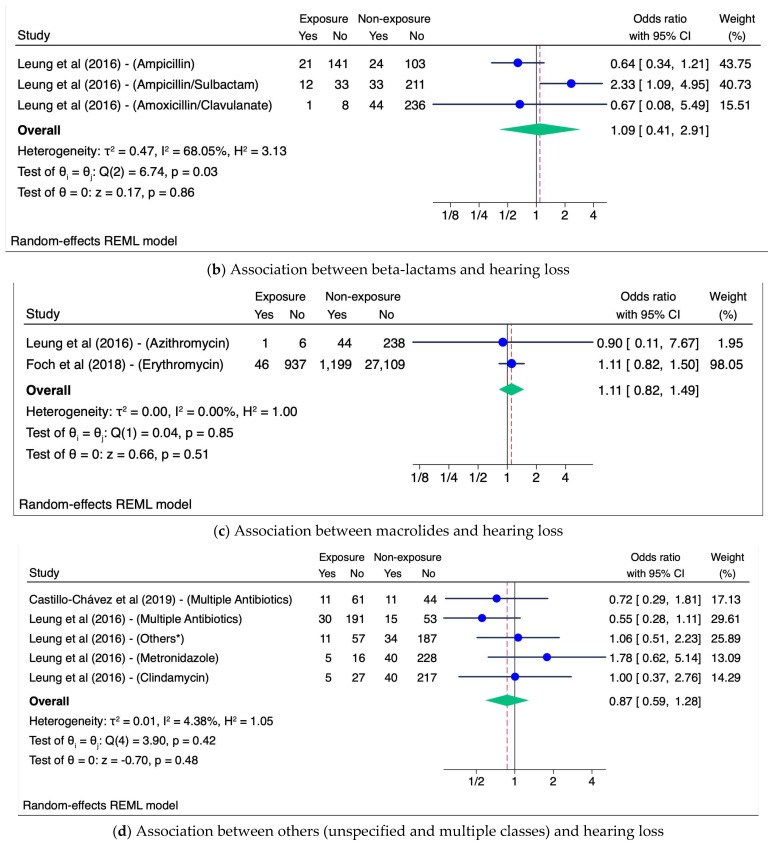
Subgroup analyses: Association between in-utero exposure to antibiotics use in pregnancy and childhood hearing loss [26,27,28,29,30,31]. Others*: sulfamethoxazole, trimethoprim, cefotetan, ceftriaxone, cephalexin, cefazolin.

Six studies were included in meta-analysis. Overall, antibiotic exposure during pregnancy was associated with a higher risk of childhood hearing loss (Pooled OR 1.2, 95% CI, 1.1–1.3, I^2^ = 15.4%) (see Figure 3). In subgroup analyses on different classes of antibiotics, aminoglycoside exposure during pregnancy was associated with higher risk of hearing loss (pooled OR = 1.2 95% CI, 1.1–1.3, I^2^ = 38.4%). However, exposure to other antibiotics classes (beta-lactams, macrolides, multiple antibiotics, and unspecified classes) during pregnancy showed no association with hearing loss (see Figure 4).

### 3.5. Descriptive Analysis on Timing, Dosage and Genetic Mutations of In-Utero Exposure to Antibiotics

Only nine studies reported the timing and dosage of antibiotic exposure during pregnancy and hearing loss in offspring with inconsistent findings. For example, Jafari et al. [38] demonstrated that gentamicin exposure during the first trimester was found in one of their child patients with hearing loss. Conway et al. [10] found two children suffering from mild unilateral hearing loss; Jones et al. [41] reported a case of an infant who did not react to sounds and experienced difficulties with speech difficulties after prenatal exposure to a daily dose of 1 g of streptomycin during the first and second trimesters of pregnancy. Similarly, Robison et al. [42] documented two cases of sensorineural hearing loss with the same exposure. However, Lyell et al. [34] showed that the dosage range of 1.5–5 mg/kg of daily gentamicin during active labor or induced labor did not cause hearing loss in newborns.

Descriptive analysis on maternal genetic mutations: One study (Xiong et al.) [30] found that genetic mutations at the GJB2 gene (235delC polymorphism) together with maternal aminoglycoside use increased the risk of congenital hearing loss in a Chinese population.

### 3.6. Risk of Bias Assessment Results

In total, 18 studies were evaluated for quality assessment using three different assessment tools, appropriate for the specific study designs (Supplemental Material Table S6). Eight studies were categorized as low risk, seven as moderate risk, and three as high risk. Among the eight cohort studies, three were low risk (scoring 3–8 points), and five were moderate risk (5–7 points). Of the four cross-sectional studies, three were high risk (2–4 points), and one was moderate (5 points). In the three case–control studies, two were low risk (7 points), and one was moderate (6 points). Two case reports scored 6 and 7 points (both low risk), and the single RCT was classified as low risk by the Cochrane RoB tool.

### 3.7. Publication Bias and Sensitivity Analysis

Egger’s test showed no strong evidence of publication bias, with a bias coefficient of 0.04 (95% CI −0.010 to 0.096, *p* = 0.105). Further analysis using the trim-and-fill method reduced the pooled effect size from 0.12 to 0.03, indicating that correction for publication bias lowered the estimated effect. However, neither the observed effect size (0.12, 95% CI −0.02 to 0.26) nor the adjusted effect size (0.03, 95% CI −0.15 to 0.22) reached statistical significance (Supplemental Table S5).

Sensitivity analysis was conducted by excluding three case reports on maternal aminoglycoside use and associated hearing loss in children, where the total sample size equaled the number of hearing loss events. After excluding these studies, the pooled prevalence was 0.03 (95% CI 0.01 to 0.05, I^2^ = 99.6%), consistent with the direction of the original pooled prevalence (Supplemental Figure S2).

## 4. Discussion

### 4.1. Key Findings

Our systematic review found that the pooled prevalence of hearing loss in children exposed to antibiotics during their mothers’ pregnancy was 0.9%. Our study result is nearly 10-fold higher than the prevalence of hearing loss in general population of the US (1–3 per 1000) [43] and Australia (1.4 per 1000) [44]. Overall, in-utero exposure to antibiotics was associated with an increased risk of childhood hearing loss (Pooled OR 1.2, 95% CI 1.1 to 1.3). Specifically, aminoglycoside exposure during pregnancy was associated with higher risk of hearing loss (Pooled OR 1.2, 95% CI 1.1 to 1.3), while other antibiotics classes (beta-lactams, macrolides, multiple antibiotics, and unspecified classes) showed no association with childhood hearing loss.

### 4.2. Interpretation in Light of Other Studies

We found that maternal aminoglycosides exposure could be a risk factor for childhood hearing loss. Aminoglycoside antibiotics are widely used in pregnancy because of their remarkable effectiveness and broad-spectrum actively against Gram-negative bacterial infections, particularly in severe conditions such as sepsis, pyelonephritis, and multidrug-resistant infections [45]. According to the Therapeutic Goods Administration (TGA) of Australia, aminoglycosides are classified as Category D antibiotics [46], indicating that there is evidence suggesting they can cause fetal harm, including ototoxicity in both the mother and the fetus. However, aminoglycoside antibiotics may be used if the potential benefits outweigh the risks in serious or life-threatening situations. Similarly, the UK Teratology Information Service (UKTIS) advises that systemic aminoglycosides should be used in pregnancy only when no safer alternatives are available. This recommendation is based on the significant risk of fetal hearing loss associated with aminoglycosides. Streptomycin has been classified as FDA Pregnancy Category D with a boxed warning, following case reports of irreversible bilateral congenital deafness linked to in-utero exposure during the first trimester [47]. This is also supported by the systematic review of Yu et al., which suggested that prenatal exposure to streptomycin may be associated with a potential risk of neonatal hearing deficits, particularly with prolonged exposure [48]. Their review reported hearing deficits in 7% (4 out of 59) of maternal streptomycin exposed infants, highlighting concerns about ototoxicity [48]. Although their focus was on streptomycin for postexposure prophylaxis of plague, the overlap with included studies strengthens the evidence for this association.

Our meta-analysis found no association between childhood hearing loss and other antibiotic classes, such as beta-lactams and macrolides. This is further supported by a systematic review by Alsowaida et al. [49], which found no significant association between macrolide exposure and hearing loss, as well as findings from Arısoy et al. [50], indicating that ototoxicity side effects of beta-lactam antibiotics are uncommon. These antibiotics fall under Categories A or B according to the TGA [46], indicating they are generally considered safer to use during pregnancy compared to Categories C or D. Category A and B antibiotics are preferred when treatment is necessary. Our findings offer reassurance regarding their safety during pregnancy from a hearing loss perspective when used appropriately.

### 4.3. Implications and Future Research Directions

This review reinforces the known ototoxicity of aminoglycosides on the developing fetal auditory system [51]. The observed associations suggest that maternal aminoglycosides exposure could be a risk marker of child hearing loss. These findings carry significant clinical implications, particularly regarding the cautious use of antibiotics during pregnancy. While the overall risk of hearing loss is low, the association with aminoglycosides emphasizes the need for judicious prescribing practices. Clinical guidelines should prioritize safer alternatives when available, balancing the benefits of treating maternal infections against the potential risks to fetal auditory development.

The findings of this review also underscore several critical research gaps. First, studies capturing precise antibiotic dosages are essential to elucidate any dose–response relationships for aminoglycoside-induced ototoxicity. Further investigation is needed into the interplay of clinical factors and co-administered treatments that may exacerbate ototoxicity. For example, conditions such as fever, hypoxia, impaired renal function, and nutritional or antioxidant deficiencies could amplify the effects of aminoglycosides. Additionally, the concomitant use of other drugs, including loop diuretics or neuromuscular blockers, may substantially increase aminoglycoside-induced auditory damage [52]. Third, the dependence on single follow-up points in the included studies limits our understanding of long-term effects. Longitudinal studies are needed to comprehensively evaluate the extended impacts of prenatal antibiotic exposure on child auditory outcomes. Finally, recent genetic studies [13] highlight the role of mitochondrial mutations, such as the m.1555A>G variant, in increasing susceptibility to aminoglycoside-induced hearing loss [53]. These findings suggest a potential role for genetic screening in pregnancy to identify at-risk individuals and enable early interventions, such as avoiding aminoglycosides or implementing enhanced fetal monitoring. However, the prevalence of aminoglycoside-related MT-RNR1 variants varies significantly across populations [54]. Therefore, understanding the distribution of genetic polymorphisms in diverse populations, particularly in multicultural settings like Australia, is essential to inform targeted clinical strategies.

### 4.4. Strengths and Limitations

Our systematic review employed a comprehensive search strategy and included a range of study designs, including randomized controlled trials, cohort studies, case–control studies, and case reports. With a large overall sample size, our study offers (1) a robust meta-analysis assessing the prevalence of hearing loss among children exposed to antibiotics in-utero; (2) a random-effects model to estimate the pooled effect sizes, presenting the associations between in-utero exposure to antibiotics and offspring hearing loss; (3) a subgroup analysis evaluating the impact of different antibiotic classes on childhood hearing loss prevalence and their effect sizes; and (4) a descriptive analysis of the timing, dosage, and genetic mutations associated with in-utero antibiotic exposure. Among the eighteen included studies, eight were classified as having a low risk of bias, and seven as having a moderate risk, highlighting the high quality of evidence and minimal selection bias.

Our review has several limitations. First, we might have underestimated the associations between in-utero exposure to antibiotics and childhood hearing loss, particularly for non-aminoglycoside antibiotics, due to the limited number of studies reporting detailed timing and dosage of exposure and the inconsistent findings among these studies. Second, the absence of information on the severity of maternal infections complicates efforts to disentangle the potential interplay between infection-related factors and ototoxicity [45,55]. Third, significant heterogeneity across included studies limited the generalizability of our findings. Our findings likely reflect variations in study design, types and timing of antibiotic exposure, definitions of hearing loss, and sample sizes. Furthermore, of the eighteen included studies, only eleven specified the audiological tests used to diagnose hearing loss, and just seven provided clear definitions of hearing loss, which varied widely. This inconsistency hindered sub-analyses based on severity or phenotype and limited cross-study comparability.

## 5. Conclusions

This study provides evidence of an increased risk of childhood hearing loss following maternal exposure to aminoglycosides, while no significant association was found for other antibiotic classes. Despite high heterogeneity and a notable prevalence of hearing loss among exposed children, these findings underscore the importance of caution when prescribing aminoglycosides during pregnancy. High-quality, long-term studies are needed to clarify if there is a dose–response relationship and inform the development of refined clinical guidelines to optimize antibiotic use in pregnant populations.

## Figures and Tables

**Table 1:** Characteristics of included studies.

Author Year	Study Design	Sample Size	Gestational Age	In-Utero Exposure to Antibiotics (Incl Dosage)	Antibiotic Use Indication	Ascertainment of Exposure	Hearing Loss Diagnosis Method	Age of Diagnosis	Hearing Loss Definition	Main Findings
Studies reporting associations between in-utero exposure to antibiotics and hearing loss in offsprings										
Tsao et al., 2025 [26]	Case–control	3596 hearing loss cases, 17,980 controls, total 21,576	<37 weeks	Gentamicin (Gent) and furosemide	N/A	3 nationwide databases with maternal and child health medical records	Diagnosis at ≥2 outpatient clinics or 1 hospital admission within 1 year	Mean ± SD All: 1.95 ± 2.33 years Cases: 1.74 ± 2.12 years Controls: 1.99 ± 2.37 years	According to ICD-9-CM 389, 794.15	Gentamicin and furosemide cOR = 1.28 (1.19–1.38) aOR 0.97 (0.89–1.06)
Tsao et al., 2023 [27]	Case–control	12,873 hearing loss cases, 64,365 controls, total 77,238	Full term	Gent and furosemide		3 nationwide databases with maternal and child health medical records	Diagnosis at ≥2 outpatient clinics or 1 hospital admission within 1 year	Mean± SD All: 2.79 ± 2.62 years Cases: 2.49 ± 2.43 years Controls:2.85 ± 2.65 years	According to ICD-9-CM 389, 794.15	Gentamicin and furosemide cOR = 1.18 (1.13–1.24) aOR 1.01 (0.96–1.07)
Castillo-Chávez et al., 2019 [28]	Case–control	22 cases, 105 controls, from one hospital	Gestational age range 30–37 weeks	Multiple: amikacin, ampicillin, cephalothin, cefalexin, cefotaxime, ceftriaxone, clindamycin (CLN), chloramphenicol, erythromycin (ERY), Gent Dosage varies between antibiotics, in 3rd trimester	Unspecified	Medical records	Transient Evoked Otoacoustic Emissions (TEOAE)	0.5 years	Hearing threshold > 30 dB HL	Multiple antibiotics OR 0.72 (0.29, 1.81)
Foch et al., 2018 [29]	Case–control	1245 cases, 28,046 controls	N/A	Gent, ERY, tobramycin, netilmicin, unspecified trimester and dosage	Unspecified	Births and pregnancy database	Abnormal hearing on health certificate	2 years	N/A	Gentamicin OR = 3.22 (0.40, 26.19) Erythromycin OR = 1.11 (0.82, 1.50), Tobramycin OR = 0, Netilmicin OR = 0
Xiong et al., 2017 [30]	Case–control	118 cases, 242 controls, from 2 hospitals	N/A	Aminoglycosides, unspecified trimester and dosage	Maternal infections unspecified	Medical records and structured questionnaires	Congenital deafness, deafness or mutism at birth	Mean ± SD Cases: 2.43 ± 1.05 years Controls: 2.57 ± 0.91 years	N/A	Aminoglycosides OR = 3.75 (1.08, 13.08)
Leung et al., 2016 [31]	Retrospective cohort	289 babies in NICU, from 2 hospitals	Gestational age < 33 weeks	Multiple: Gent, ERY, azithromycin, ampicillin/sulbactam, amoxicillin/clavulanate, metronidazole, CLN, and others% (dosage or trimester of exposure not specified)	Group B Streptococcus Latency Suspected chorioamnionitis Maternal infection	Medical record	automated auditory brainstem response (AABR)	N/A	Hearing loss > 30 dB HL	ORs: Multiple abx 0.55 (0.28, 1.11), Others abx group 1.06 (0.51, 2.23), Gent 1.82 (0.68, 4.85), Azithromycin 0.90 (0.11, 7.67), Ampicillin 0.64 (0.34, 1.21), Ampicillin/sulbactam 2.33 (1.09, 4.95), Amoxicillin/clavulanate 0.67 (0.08, 5.49) Metronidazole 1.78 (0.62, 5.14) CLN 1.00 (0.37, 2.76)
Studies reporting the number of hearing loss cases in children with mothers exposed to antibiotics in pregnancy										
Patatt et al., 2021 [32]	Cohort	527 newborns from 2 sites	Gestational age: 38.4–38.6 weeks	CLN + Quinine, 3rd trimester		Interview and medical records	TEOAE and/or Automated Brainstem Auditory Evoked Potential (A-BAEP)	N/A	N/A	No babies developed hearing loss
Stadio et al., 2019 [33]	Prospective cohort	153 babies in NICU	Gestational age mean 33 weeks, range: 23–42 weeks	Unspecified antibiotics	Maternal infections #	Medical records,	TEOAE and AABR	N/A	Moderate (41–55 dB HL) Profound (>91 dB HL)	No babies developed hearing loss
Lyell et al., 2010 [34]	Randomized controlled trial	125 mothers from one California hospital	N/A	Group 1: Daily Gent * Group 2: 8-Hour Gentamicin ** For Caesarean Birth: CLN: 900 mg every 8 h (3 doses in total). Gestation: 32–42 wks.	Intrapartum Chorioamnionitis	Clinical trial and exam	AABR	At birth	Failing hearing screen	No babies developed hearing loss
Saunders et al., 2009 [35]	Cross-sectional	31 cases, from an otolaryngology/audiology clinic	N/A	Gent, unspecified timing and dosage	Meningitis Perinatal distress	Questionnaire	Air and bone conduction audiometry at 500 Hz, 1000 Hz, 2000 Hz, 4000 Hz.	Before 18 years old	Mild (21–40 dB) Moderate (41–60 dB) Severe (61–80 dB) Profound (>80 dB)	Only 2 cases with bilateral profound hearing loss were exposed to abx during pregnancy *
Saunders et al., 2007 [36]	Cross-sectional	96 cases, from an otolaryngology/audiology clinic	N/A	Gent, unspecified timing and dosage	Unspecified	Questionnaire	Air and bone conduction audiometry	During 1st 18 years of life	Mild (21–40 dB) Moderate (41–60 dB) Severe (61–80 dB) Profound (>80 dB)	Only 2 cases with bilateral profound hearing loss were exposed to abx during pregnancy
Kirkwood et al., 2007 [37]	Cohort	40 babies, from one hospital	N/A	Gent, at least one dose, intravenous, mean dose ± SD: 764 mg ± 600 mg for mean duration 2.7 ± 2.3 days, at average gestational age of 28 ± 6 weeks	Maternal infections unspecified	Pharmacy records and chart review	TEOAE and AABR	1st 3 days after birth	N/A	Out of 40 babies exposed, none developed hearing loss
Jafari et al., 2007 [38]	Cross-sectional	86 children from Newsha Aural Rehabilitation Center (all children had hearing loss)	N/A	Gent (1st trimester), Kanamycin (3rd trimester)	Unspecified	Questionnaire	Pure tone audiometry	Average age of diagnosis = 15.2 ± 9.3 months (range 1–36 months)	Bilateral profound hearing loss ≥ 91 dB HL	2 cases with mothers who took abx during pregnancy (1 Gent, 1 kanamycin)
Ganga et al., 1991 [39]	Cohort	78 students from a government school for deaf children	N/A	Streptomycin (STR), unspecified trimester and dosage	Maternal infections: syphilis, chickenpox	Interview	Failing Rinne and Weber tests (air/bone conduction threshold)	N/A	N/A	2 out of the 78 students had been exposed to STR
Donald et al., 1991 [40]	Cohort	30 children from Brooklyn hospital and nearby clinics	N/A	STR, duration ranged between 0.25–2 months, various trimesters throughout pregnancy	Tuberculosis	Hospital/clinic records	Failing unspecified hearing tests	13–48 months of age	N/A	Severe unilateral deafness in 1 child exposed during 1st trimester
Jones 1973 [41]	Case report	1 case	N/A	Kanamycin (1 g IM per day, total dose 4.5 g, at 28 weeks gestation	Urinary traction infections	Clinical records and examinations	Unspecified audiologic tests. No speech development, no response to sounds in the environment or outside field of vision	1 and 3 years of age	N/A	1 case passed hearing tests but did not react to sounds and difficulties with speech
Conway et al., 1965 [10]	Case series	17 children, from one London hospital	N/A	1 g per day/3-days/biweekly/6-days STR or myxtamycin (STR + dihydrostreptomycin), total doses ranged 29–202 g, varying trimesters of exposure	Tuberculosis	Case records	Air-conduction audiometry	Tests done on children during study, age range between 6–13 years	Mild > 20 dB HL, Moderate > 40 dB HL, Severe > 60 dB HL	4 unilateral high-tone hearing loss cases (3 mild, 1 severe) had mothers who took abx 1 g/day during pregnancy
Robinson et al., 1964 [42]	Case report	2 cases	N/A	Case 1: 1 g STR biweekly between weeks 6-14 of pregnancy Case 2: 1 g STR biweekly during last 4 months of pregnancy	Tuberculosis	Case records	N/A	Case 1: diagnosed at ~4 years Case 2: diagnosed at ~2–3 years old	N/A	Case 1: severe bilateral SNHL, Case 2: severe bilateral SNHL

Note: TEOAE, Transient Evoked Otoacoustic Emissions. A-BAEP, Automated Brain-stem Auditory Evoked Potential. AABR, Automated Auditory Brainstem Responses. SNHL: Sensorineural hearing loss; * Daily Gentamicin: Gentamicin: 5 mg/kg once daily. Placebo (Saline): Given at 8 and 16 h. Ampicillin: 2 g every 6 h (4 doses in total). ** 8-Hour Gentamicin: Gentamicin: 2 mg/kg loading dose, followed by 1.5 mg/kg at 8 and 16 h. Ampicillin: 2 g every 6 h (4 doses in total). # *Cytomegalovirus*, *Toxoplasma*, Rubeola, *Candida albicans*, *E. coli*, *Chlamydia*, *Syphilis*, *Mycoplasma* and Hepatitis C virus. % sulfamethoxazole and trimethoprim, cefotetan, ceftriaxone, cephalexin, cefazolin.

## Data Availability

Not applicable to this article as no datasets were generated or analyses during the current study.

## Supplementary Materials

The following supporting information can be downloaded at: https://www.mdpi.com/article/10.3390/children12030356/s1, Table S1. List of antibiotics. Table S2. Medline search strategy. Table S3. Embase search strategy. Table S4. PubMed search strategy. Table S5. Publication bias. Table S6. Summary of quality assessment for the included studies.. Table S7. Summary of included studies. Figure S1. Pooled prevalence divided by different antibiotic classes. Figure S2. Sensitivity analysis on pooled prevalence.

## References

[1] Deafness and Hearing Loss. https://www.who.int/news-room/fact-sheets/detail/deafness-and-hearing-loss.

[2] World Health Organization (2017). Global Costs of Unaddressed Hearing Loss and Cost-Effectiveness of Interventions: A WHO Report.

[3] Tomblin J.B., Harrison M., Ambrose S.E., Walker E.A., Oleson J.J., Moeller M.P. (2015). Language outcomes in young children with mild to severe hearing Loss. Ear Hear..

[4] Wang J., Quach J., Sung V., Carew P., Edwards B., Grobler A., Gold L., Wake M. (2019). Academic, behavioural and quality of life outcomes of slight to mild hearing loss in late childhood: A population-based study. Arch. Dis. Child.

[5] Mathers C., Smith A., Concha M. (2000). Global burden of hearing loss in the year 2000. Glob. Burd. Dis..

[6] Bookstaver P.B., Bland C.M., Griffin B., Stover K.R., Eiland L.S., McLaughlin M. (2015). A Review of Antibiotic Use in Pregnancy. Pharmacotherapy.

[7] Korpela K., Salonen A., Saxen H., Nikkonen A., Peltola V., Jaakkola T., de Vos W., Kolho K.L. (2020). Antibiotics in early life associate with specific gut microbiota signatures in a prospective longitudinal infant cohort. Pediatr. Res..

[8] Arboleya S., Sánchez B., Milani C., Duranti S., Solís G., Fernández N., de los Reyes-Gavilán C.G., Ventura M., Margolles A., Gueimonde M. (2015). Intestinal microbiota development in preterm neonates and effect of perinatal antibiotics. J. Pediatr..

[9] Volkova A., Ruggles K., Schulfer A., Gao Z., Ginsberg S.D., Blaser M.J. (2021). Effects of early-life penicillin exposure on the gut microbiome and frontal cortex and amygdala gene expression. iScience.

[10] Conway N., Birt B.D. (1965). Streptomycin in Pregnancy: Effect on the Foetal Ear. Br. Med. J..

[11] Miller J.E., Wu C., Pedersen L.H., de Klerk N., Olsen J., Burgner D.P. (2018). Maternal antibiotic exposure during pregnancy and hospitalization with infection in offspring: A population-based cohort study. Int. J. Epidemiol..

[12] Hu Y.J., Wang J., Harwell J.I., Wake M. (2021). Association of in utero antibiotic exposure on childhood ear infection trajectories: Results from a national birth cohort study. J. Paediatr. Child Health.

[13] Bitner-Glindzicz M., Rahman S., Chant K., Marlow N. (2014). Gentamicin, genetic variation and deafness in preterm children. BMC Pediatr..

[14] Page M.J., McKenzie J.E., Bossuyt P.M., Boutron I., Hoffmann T.C., Mulrow C.D., Shamseer L., Tetzlaff J.M., Akl E.A., Brennan S.E. (2021). The PRISMA 2020 statement: An updated guideline for reporting systematic reviews. BMJ Clin. Res. Ed..

[15] McHugh M.L. (2012). Interrater reliability: The kappa statistic. Biochem. Med..

[16] Munn Z., Barker T.H., Moola S., Tufanaru C., Stern C., McArthur A., Stephenson M., Aromataris E. (2020). Methodological quality of case series studies: An introduction to the JBI critical appraisal tool. JBI Evid. Synth..

[17] Wells G.A., Shea B., O’Connell D., Peterson J., Welch V., Losos M., Tugwell P. (2000). The Newcastle-Ottawa Scale (NOS) for Assessing the Quality of Nonrandomised Studies in Meta-Analyses.

[18] Sterne J.A.C., Savović J., Page M.J., Elbers R.G., Blencowe N.S., Boutron I., Cates C.J., Cheng H.Y., Corbett M.S., Eldridge S.M. (2019). RoB 2: A revised tool for assessing risk of bias in randomised trials. BMJ Clin. Res. Ed..

[19] McGuire T.M., Smith J., Del Mar C. (2015). The match between common antibiotics packaging and guidelines for their use in Australia. Aust. N. Z. J. Public Health.

[20] Friedrich J.O., Adhikari N.K., Beyene J. (2007). Inclusion of zero total event trials in meta-analyses maintains analytic consistency and incorporates all available data. BMC Med. Res. Methodol..

[21] Yiengprugsawan V., Hogan A., Strazdins L. (2013). Longitudinal analysis of ear infection and hearing impairment: Findings from 6-year prospective cohorts of Australian children. BMC Pediatr..

[22] DerSimonian R., Laird N. (1986). Meta-analysis in clinical trials. Control. Clin. Trials.

[23] Kanters S. (2022). Fixed-and random-effects models. Meta-Res. Methods Protoc..

[24] Deeks J.J., Higgins J.P., Altman D.G., on behalf of the Cochrane Statistical Methods Group (2019). Analysing data and undertaking meta-analyses. Cochrane Handbook for Systematic Reviews of Interventions.

[25] Egger M., Smith G.D., Schneider M., Minder C. (1997). Bias in meta-analysis detected by a simple, graphical test. BMJ Clin. Res. Ed..

[26] Tsao P.C., Lin H.C., Shen S.P., Chang Y.C. (2025). Exploring predisposing factors of hearing loss in prematurely born children: A nationwide case-control study. Pediatr. Neonatol..

[27] Tsao P.C., Lin H.C., Chiu H.Y., Chang Y.C. (2023). Maternal, Perinatal, and Postnatal Predisposing Factors of Hearing Loss in Full-Term Children: A Matched Case-Control Study. Neonatology.

[28] Castillo-Chavez A.M., Monroy-Torres R., Hernandez Gonzalez V.H. (2019). Association between food insecurity and perinatal risk factors with hearing problems in preterm birth. Nutr. Hosp..

[29] Foch C., Araujo M., Weckel A., Damase-Michel C., Montastruc J.L., Benevent J., Durrieu G., Lacroix I. (2018). In utero drug exposure and hearing impairment in 2-year-old children A case-control study using the EFEMERIS database. Int. J. Pediatr. Otorhinolaryngol..

[30] Xiong Y., Zhong M., Chen J., Yan Y.L., Lin X.F., Li X. (2017). Effect of GJB2 235delC and 30-35delG genetic polymorphisms on risk of congenital deafness in a Chinese population. Genet. Mol. Res..

[31] Leung J.C., Cifra C.L., Agthe A.G., Sun C.C., Viscardi R.M. (2016). Antenatal factors modulate hearing screen failure risk in preterm infants. Arch. Dis. Child. Fetal Neonatal Ed..

[32] Patatt F.S.A., Sampaio A.L.L., Tauil P.L., Oliveira C. (2021). Hearing of neonates without risk indicators for hearing loss and use of antimalarial drugs during pregnancy: A historical cohort study in the Northern Region of Brazil. Braz. J. Otorhinolaryngol..

[33] Stadio A.D., Molini E., Gambacorta V., Giommetti G., Volpe A.D., Ralli M., Lapenna R., Trabalzini F., Ricci G. (2019). Sensorineural Hearing Loss in Newborns Hospitalized in Neonatal Intensive Care Unit: An Observational Study. Int. Tinnitus J..

[34] Lyell D.J., Pullen K., Fuh K., Zamah A.M., Caughey A.B., Benitz W., El-Sayed Y.Y. (2010). Daily compared with 8-hour gentamicin for the treatment of intrapartum chorioamnionitis: A randomized controlled trial. Obstet. Gynecol..

[35] Saunders J.E., Greinwald J.H., Vaz S., Guo Y. (2009). Aminoglycoside ototoxicity in Nicaraguan children: Patient risk factors and mitochondrial DNA results. Otolaryngol. Head Neck Surg..

[36] Saunders J.E., Vaz S., Greinwald J.H., Lai J., Morin L., Mojica K. (2007). Prevalence and etiology of hearing loss in rural Nicaraguan children. Laryngoscope.

[37] Kirkwood A., Harris C., Timar N., Koren G. (2007). Is gentamicin ototoxic to the fetus?. J. Obs. Gynaecol. Can..

[38] Jafari Z., Malayeri S., Ashayeri H. (2007). The ages of suspicion, diagnosis, amplification, and intervention in deaf children. Int. J. Pediatr. Otorhinolaryngol..

[39] Ganga N., Rajagopal B., Rajendran S., Padmanabhan A.S. (1991). Deafness in children—An analysis. Indian. Pediatr..

[40] Donald P.R., Doherty E., Van Zyl F.J. (1991). Hearing loss in the child following streptomycin administration during pregnancy. Cent. Afr. J. Med..

[41] Jones H.C. (1973). Intrauterine ototoxicity. A case report and review of literature. J. Natl. Med. Assoc..

[42] Robinson G.C., Cambon K.G. (1964). Hearing Loss in Infants of Tuberculous Mothers Treated with Streptomycin during Pregnancy. N. Engl. J. Med..

[43] Centers for Disease Control and Prevention (2020). Data and Statistics About Hearing Loss in Children.

[44] Sharma R., Gu Y., Sinha K., Ching T.Y., Marnane V., Gold L., Wake M., Wang J., Parkinson B. (2022). An economic evaluation of Australia’s Newborn hearing screening program: A within-study cost-effectiveness analysis. Ear Hear..

[45] Megli C.J., Coyne C.B. (2022). Infections at the maternal–fetal interface: An overview of pathogenesis and defence. Nat. Rev. Microbiol..

[46] Prescribing Medicines in Pregnancy Database. https://www.tga.gov.au/prescribing-medicines-pregnancy-database.

[47] Heikkilä A.M. (1993). Antibiotics in pregnancy—A prospective cohort study on the policy of antibiotic prescription. Ann. Med..

[48] Yu P.A., Tran E.L., Parker C.M., Kim H.J., Yee E.L., Smith P.W., Russell Z., Nelson C.A., Broussard C.S., Yu Y.C. (2020). Safety of Antimicrobials During Pregnancy: A Systematic Review of Antimicrobials Considered for Treatment and Postexposure Prophylaxis of Plague. Clin. Infect. Dis..

[49] Alsowaida Y.S., Almulhim A.S., Oh M., Erstad B., Abraham I. (2021). Sensorineural hearing loss with macrolide antibiotics exposure: A meta-analysis of the association. Int. J. Pharm. Pr..

[50] Arısoy A.E., Arısoy E.S., Muluk N.B., Cingi C., Correa A.G. (2023). Hearing Loss in Congenital, Neonatal and Childhood Infections.

[51] Dai C.F., Mangiardi D., Cotanche D.A., Steyger P.S. (2006). Uptake of fluorescent gentamicin by vertebrate sensory cells in vivo. Hear. Res..

[52] Garinis A.C., Kemph A., Tharpe A.M., Weitkamp J.H., McEvoy C., Steyger P.S. (2018). Monitoring neonates for ototoxicity. Int. J. Audiol..

[53] Dehne N., Rauen U., de Groot H., Lautermann J. (2002). Involvement of the mitochondrial permeability transition in gentamicin ototoxicity. Hear. Res..

[54] McDermott J.H., Wolf J., Hoshitsuki K., Huddart R., Caudle K.E., Whirl-Carrillo M., Steyger P.S., Smith R.J.H., Cody N., Rodriguez-Antona C. (2022). Clinical Pharmacogenetics Implementation Consortium Guideline for the Use of Aminoglycosides Based on MT-RNR1 Genotype. Clin. Pharmacol. Ther..

[55] Ross E.J., Graham D.L., Money K.M., Stanwood G.D. (2015). Developmental Consequences of Fetal Exposure to Drugs: What We Know and What We Still Must Learn. Neuropsychopharmacology.

