# Supplementary material for: Association Between In-Utero Exposure to Antibiotics and Offspring’s Hearing Loss: A Systematic Review and Meta-Analysis
Source: Children (Basel). 2025 Mar 13;12(3):356. doi: 10.3390/children12030356 (PMC11940907; doi:10.3390/children12030356)
Supplement: Supplementary file 1 [file children-12-00356-s001.zip › children-3483651-supplementary.pdf]

Supplementary Materials:

Table S1. List of antibiotics

**Antibiotics Prescribed during Pregnancy (Australia, United States, UK)**			
**ANTIBIOTIC DRUG CLASSES/TYPES**<br>Beta-Lactam, Carbapenem, Cephalosporin, Glycopeptide, Lipoglycopeptide, Macrolide, Quinolone, Fluoroquinolone, Sulfonamide/Sulphonamide, Streptogramin, Tetracycline, Aminoglycoside			
**ANTIBIOTIC DRUG NAMES**			
alatrofloxacin	colistin	minocyn	ticarcillin
amikacin	colistimethate	moxifloxacin	tigecycline
amoxicillin	colistin IV with colistimethate	mupirocin	tinidazole
ampicillin	cotrimoxazole	nalidixic acid	topicycline
atovaquone	dalbavancin	nitrofurantoin	trimethoprim
azithromycin	dalfopristin with quinupristin	nitroimidazole	trovafloxacin
azlocillin	daptomycin	norfloxacin	vancomycin
aztreonam	demeclocycline	ofloxacin	vibramycin
bactrim	dicloxacillin	omadacycline	
bedaquiline	doripenem	oritavancin	
benznidazole	doxycycline	oseltamivir	
benzathine penicillin	doxymycin	oxazolidinone	
benzylpenicillin	enoxacin	oxytetracycline	
besifloxacin	eravacycline	ozenoxacin	
cefaclor	ertapenem	pentamidine	
cefalexin	erythromycin	permethrin	
cefalotin	fexinidazole	phenoxymethylpenicillin	
cefamandole	fidaxomicin	piperacillin	
cefazolin	finafloxacin	piperacillin with tazobactam	
cefepime	fleroxacin	plazomicin	
cefodizime	flucloxacillin	procaine benzylpenicillin	
cefotaxime	fosfomycin trometamol	procaine penicillin	
cefotetan	fusidic acid	relebactam	
cefoxitin	gatifloxacin	rifampin	
cefpirome	gentamicin/gentamycin	rifampicin	
cefpodoxime	imipenem with cilastatin	rifaximin	
ceftaroline fosamil	levofloxacin	rifamycin	
ceftazidime	lincomycin	rolitetracycline	
ceftolozane/tazobactam	linezolid	roxithromycin	
ceftriaxone	loracarbef	sarecycline	
cefuroxime	lymecycline	spectinomycin	
chinolone	macrotetrolide	spiramycin	
chloramphenicol	meciclin	streptomycin/streptomicin	
chlortetracycline	mexacine/mexocine	sulfamethoprim	
chloromycetin	meropenem	sulfamethoxazole	
ciprofloxacin	metacycline	sulfaprim	
clarithromycin	methacycline	tedizolid	
clavulanate	metronidazole	telavancin	
clavulanic acid	mezlocillin	teicoplanin	
clindamycin	micafungin	terramycin	
cloxacillin	minocycline	tetracyn	

Table S2. Medline Search Strategy

**1**	exp Anti-Bacterial Agents/ or exp Tetracyclines/ or exp Amoxicillin/ or exp Macrolides/ or exp beta-lactams/ or exp fluoroquinolones/ or exp Aminoglycosides/ or exp Antibiotic Prophylaxis/
**2**	anti-infective agents/ or lactams/ or quinolones/ or sulfamethoxazole/ or trimethoprim, sulfamethoxazole drug combination/ or Trimethoprim/
**3**	(antibiotic or antibiotics or ((antibacterial* or anti-bacterial* or bacteriocid* or antimicrobial* or anti-microbial* or antimycrobial* or anti-mycrobial* or antiinfective or anti-infective) adj1 agent*)).tw,kf.
**4**	(aminoglycoside* or penicillin* or amoxcin* or amoxcillin* or amoxiclin* or amoxillin* or amoxycillin* or amoxicillin* or carbapenem* or cephalosporin* or macrolide* or macrotetrolide* or quinolone* or chinolone* or tetracyclin* or chlortetracyclin* or chlortetracylin* or demeclocyclin* or meciclin* or mexacine* or mexocine* or doxycyclin* or doxymycin* or metacyclin* or methacyclin* or minocyclin* or minocyn* or oxytetracyclin* or rolitetracyclin* or tetracyn* or topicyclin* or trimethoprim* or trimetoprim* or trimethoprin* or fluoroquinolone* or cotrimoxazole* or bactrim* or sulfamethoprim* or sulfamethoxazole* or sulfaprim* or beta-lactam* or Glycopeptide* or Tigecycline* or Streptogramin* or Linezolid* or Colistin* or Sulfonamide* or sulphonamide* or Nitrofurantoin* or Fosfomycin* or alatrofloxacin* or amikacin* or ampicillin* or amoxicillin-clavulanate* or clavulanate* or clavulanic* or atovaquone* or azithromycin* or azlocillin* or aztreonam* or benzylpenicillin* or benzathine-benzylpenicillin* or cefaclor* or cefalexin* or cephalexin* or cefalotin* or cephalothin* or cefamandole* or cefazolin* or cephazolin* or cefepime* or cefodizime* or cefotaxime* or cefotetan* or cefoxitin* or cefpirome* or cefpodoxime* or ceftaroline* or ceftazidime* or ceftolozane* or ceftriaxone* or cefuroxime* or chloramphenicol* or ciprofloxacin* or clarithromycin* or clindamycin* or cloxacillin* or colistimethate* or colistin-IV-with-colistimethate* or dalfopristin* or quinupristin* or daptomycin* or dicloxacillin* or doripenem* or enoxacin* or ertapenem* or erythromycin* or fidaxomicin* or fleroxacin* or flucloxacillin* or fosfomycin-trometamol* or fusidic-acid* or gatifloxacin* or gentamicin* or gentamycin* or imipenem* or lincomycin* or loracarbef* or meropenem* or metronidazole* or mezlocillin* or micafungin* or moxifloxacin* or nalidixic-acid* or norfloxacin* or ofloxacin* or oseltamivir* or pentamidine* or permethrin* or phenoxymethylpenicillin* or piperacillin* or procaine-benzylpenicillin* or rifampicin* or rifaximin* or roxithromycin* or spectinomycin* or tazobactam* or teicoplanin* or ticarcillin* or tinidazole* or trovafloxacin* or vancomycin* or nitroimidazole* or lipoglycopeptide* or oxazolidinone* or rifamycin* or sarecycline* or relebactam* or rifampin* or benznidazole* or besifloxacin* or dalbavancin* or fexinidazole* or oritavancin* or omadacycline* or bedaquiline* or tedizolid* or telavancin* or ozenoxacin* or eravacycline* or finafloxacin* or plazomicin* or chloromycetin* or vibramycin* or levofloxacin* or lymecycline* or terramycin* or spiramycin*).tw,kf.
**5**	1 or 2 or 3 or 4
**6**	Pregnancy/ or Pregnant Women/ or Maternal Exposure/ or ((gestation* or in-utero or mother* or maternal* or antenatal* or prenatal*) and ("use" or expose* or exposure* or usage)).tw,kf.
**7**	Prenatal Exposure Delayed Effects/ or Infectious Disease Transmission, Vertical/ or Maternal-Fetal Exchange/
**8**	(newborn* or new-born* or baby or babies or neonat* or neo-nat* or infan* or fetus* or fetus* or fetal or foetal or toddler* or child* or offspring* or toddler* or pre-schooler* or preschooler* or kindergarten or boy or boys or girl or girls or adolescen* or pediatric* or paediatric* or youth* or teen or teens or teenage*).af.
**9**	exp hearing loss/ or deafness/ or *Cochlear Implantation/ or *Cochlear Implants/ or *hearing aids/ or Hearing tests/ or audiometry/ or audiometry, evoked response/ or audiometry, pure-tone/ or exp audiometry, speech/ or (hearing* or deaf* or hard-of-hearing* or audiometr* or hearing-aid* or cochlear-implant* or deaf-and-hard-of-hearing).tw,kf.
**10**	5 and (6 or 7) and 8 and 9
**11**	limit 10 to (comment or editorial or guideline or letter or practice guideline)
**12**	10 not 11

Table S3. Embase Search Strategy

**1**	exp antibiotic agent/ or exp antiinfective agent/ or exp antibiotic prophylaxis/ or exp leprostatic agent/ or exp beta lactam antibiotic/ or exp beta lactamase inhibitor/ or tetracycline/ or amoxicillin/ or sulfamethoxazole/ or cotrimoxazole/ or trimethoprim/ or quinolone derivative/ or macrolide/ or aminoglycoside antibiotic agent/
**2**	(antibiotic or antibiotics or ((antibacterial* or anti-bacterial* or bacteriocid* or antimicrobial* or anti-microbial* or antimycrobial* or anti-mycrobial* or antiinfective or anti-infective) adj1 agent*)).tw,kf,dq.
**3**	(aminoglycoside* or penicillin* or amoxcin* or amoxcillin* or amoxiclin* or amoxillin* or amoxycillin* or amoxicillin* or carbapenem* or cephalosporin* or macrolide* or macrotetrolide* or quinolone* or chinolone* or tetracyclin* or chlortetracyclin* or chlortetracylin* or demeclocyclin* or meciclin* or mexacine* or mexocine* or doxycyclin* or doxymycin* or metacyclin* or methacyclin* or minocyclin* or minocyn* or oxytetracyclin* or rolitetracyclin* or tetracyn* or topicyclin* or trimethoprim* or trimetoprim* or trimethoprin* or fluoroquinolone* or cotrimoxazole* or bactrim* or sulfamethoprim* or sulfamethoxazole* or sulfaprim* or beta-lactam* or Glycopeptide* or Tigecycline* or Streptogramin* or Linezolid* or Colistin* or Sulfonamide* or sulphonamide* or Nitrofurantoin* or Fosfomycin* or alatrofloxacin* or amikacin* or ampicillin* or amoxicillin-clavulanate* or clavulanate* or clavulanic* or atovaquone* or azithromycin* or azlocillin* or aztreonam* or benzylpenicillin* or benzathine-benzylpenicillin* or cefaclor* or cefalexin* or cephalexin* or cefalotin* or cephalothin* or cefamandole* or cefazolin* or cephazolin* or cefepime* or cefodizime* or cefotaxime* or cefotetan* or cefoxitin* or cefpirome* or cefpodoxime* or ceftaroline* or ceftazidime* or ceftolozane* or ceftriaxone* or cefuroxime* or chloramphenicol* or ciprofloxacin* or clarithromycin* or clindamycin* or cloxacillin* or colistimethate* or colistin-IV-with-colistimethate* or dalfopristin* or quinupristin* or daptomycin* or dicloxacillin* or doripenem* or enoxacin* or ertapenem* or erythromycin* or fidaxomicin* or fleroxacin* or flucloxacillin* or fosfomycin-trometamol* or fusidic-acid* or gatifloxacin* or gentamicin* or gentamycin* or imipenem* or lincomycin* or loracarbef* or meropenem* or metronidazole* or mezlocillin* or micafungin* or moxifloxacin* or nalidixic-acid* or norfloxacin* or ofloxacin* or oseltamivir* or pentamidine* or permethrin* or phenoxymethylpenicillin* or piperacillin* or procaine-benzylpenicillin* or rifampicin* or rifaximin* or roxithromycin* or spectinomycin* or tazobactam* or teicoplanin* or ticarcillin* or tinidazole* or trovafloxacin* or vancomycin* or nitroimidazole* or lipoglycopeptide* or oxazolidinone* or rifamycin* or sarecycline* or relebactam* or rifampin* or benznidazole* or besifloxacin* or dalbavancin* or fexinidazole* or oritavancin* or omadacycline* or bedaquiline* or tedizolid* or telavancin* or ozenoxacin* or eravacycline* or finafloxacin* or plazomicin* or chloromycetin* or vibramycin* or levofloxacin* or lymecycline* or terramycin* or spiramycin*).tw,kf,dq.
**4**	1 or 2 or 3
**5**	pregnancy/ or pregnant woman/ or maternal exposure/ or ((gestation* or in-utero or mother* or maternal* or antenatal* or prenatal*) and ("use" or expose* or exposure* or usage)).tw,kf,dq.
**6**	prenatal exposure/ or maternal fetal transmission/ or mother to child transmission/ or vertical transmission/ or fetomaternal transfusion/
**7**	(newborn* or new-born* or baby or babies or neonat* or neo-nat* or infan* or fetus* or fetus* or fetal or foetal or toddler* or child* or offspring* or toddler* or pre-schooler* or preschooler* or kindergarten or boy or boys or girl or girls or adolescen* or pediatric* or paediatric* or youth* or teen or teens or teenage*).af.
**8**	exp hearing impairment/ or hearing impaired person/ or cochlea prosthesis/ or hearing aid/ or audiometry/ or hearing test/ or evoked response audiometry/ or pure tone audiometry/ or speech audiometry/ or (hearing* or deaf* or hard-of-hearing* or audiometr* or hearing-aid* or cochlear-implant* or deaf-and-hard-of-hearing).tw,kf,dq.
**9**	4 and (5 or 6) and 7 and 8
**10**	limit 9 to (conference abstract or conference paper or "conference review" or editorial or letter)
**11**	9 not 10

Table S4. PubMed Search Strategy

**#1 All Fields**	"antibiotic" OR "antibiotics" OR "prophylaxis" OR "prophylactic" OR (("antibacterial" OR "antibacterials" OR "anti-bacterial" OR "ant- bacterials" OR "bacteriocide" OR "bacteriocides" OR "antimicrobial" OR "antimicrobials" OR "anti-microbial" OR "anti-microbials" OR "antimycrobial" OR "antimycrobials" OR "anti-mycrobial" OR "anti-mycrobials" OR "antiinfective" OR "antiinfectives" OR "anti-infective" OR "anti-infectives" OR "leprostatic" OR "leprostatics" OR "prophylactic") AND ("agent" OR "agents"))
**#2 All Fields**	"aminoglycoside*" OR "penicillin*" OR "amoxcin*" OR "amoxcillin*" OR "amoxiclin*" OR "amoxillin*" OR "amoxycillin*" OR "amoxicillin*" OR "carbapenem*" OR "cephalosporin*" OR "macrolide*" OR "macrotetrolide*" OR "quinolone*" OR "chinolone*" OR "tetracyclin*" OR "chlortetracyclin*" OR "chlortetracylin*" OR "demeclocyclin*" OR "meciclin*" OR "mexacine*" OR "mexocine*" OR "doxycyclin*" OR "doxymycin*" OR "metacyclin*" OR "methacyclin*" OR "minocyclin*" OR "minocyn*" OR "oxytetracyclin*" OR "rolitetracyclin*" OR "tetracyn*" OR "topicyclin*" OR "trimethoprim*" OR "trimetoprim*" OR "trimethoprin*" OR "fluoroquinolone*" OR "cotrimoxazole*" OR "bactrim*" OR "sulfamethoprim*" OR "sulfamethoxazole*" OR "sulfaprim*" OR "beta-lactam*" OR "Glycopeptide*" OR "Tigecycline*" OR "Streptogramin*" OR "Linezolid*" OR "Colistin*" OR "Sulfonamide*" OR "sulphonamide*" OR "Nitrofurantoin*" OR "Fosfomycin*" OR "alatrofloxacin*" OR "amikacin*" OR "ampicillin*" OR "amoxicillin-clavulanate*" OR "clavulanate*" OR "clavulanic*" OR "atovaquone*" OR "azithromycin*" OR "azlocillin*" OR "aztreonam*" OR "benzylpenicillin*" OR "benzathine-benzylpenicillin*" OR "cefaclor*" OR "cefalexin*" OR "cephalexin*" OR "cefalotin*" OR "cephalothin*" OR "cefamandole*" OR "cefazolin*" OR "cephazolin*" OR "cefepime*" OR "cefodizime*" OR "cefotaxime*" OR "cefotetan*" OR "cefoxitin*" OR "cefpirome*" OR "cefpodoxime*" OR "ceftaroline*" OR "ceftazidime*" OR "ceftolozane*" OR "ceftriaxone*" OR "cefuroxime*" OR "chloramphenicol*" OR "ciprofloxacin*" OR "clarithromycin*" OR "clindamycin*" OR "cloxacillin*" OR "colistimethate*" OR "colistin-IV-with-colistimethate*" OR "dalfopristin*" OR "quinupristin*" OR "daptomycin*" OR "dicloxacillin*" OR "doripenem*" OR "enoxacin*" OR "ertapenem*" OR "erythromycin*" OR "fidaxomicin*" OR "fleroxacin*" OR "flucloxacillin*" OR "fosfomycin-trometamol*" OR "fusidic-acid*" OR "gatifloxacin*" OR "gentamicin*" OR "gentamycin*" OR "imipenem*" OR "lincomycin*" OR "loracarbef*" OR "meropenem*" OR "metronidazole*" OR "mezlocillin*" OR "micafungin*" OR "moxifloxacin*" OR "nalidixic-acid*" OR "norfloxacin*" OR "ofloxacin*" OR "oseltamivir*" OR "pentamidine*" OR "permethrin*" OR "phenoxymethylpenicillin*" OR "piperacillin*" OR "procaine-benzylpenicillin*" OR "rifampicin*" OR "rifaximin*" OR "roxithromycin*" OR "spectinomycin*" OR "tazobactam*" OR "teicoplanin*" OR "ticarcillin*" OR "tinidazole*" OR "trovafloxacin*" OR "vancomycin*" OR "nitroimidazole*" OR "lipoglycopeptide*" OR "oxazolidinone*" OR "rifamycin*" OR "sarecycline*" OR "relebactam*" OR "rifampin*" OR "benznidazole*" OR "besifloxacin*" OR "dalbavancin*" OR "fexinidazole*" OR "oritavancin*" OR "omadacycline*" OR "bedaquiline*" OR "tedizolid*" OR "telavancin*" OR "ozenoxacin*" OR "eravacycline*" OR "finafloxacin*" OR "plazomicin*" OR "chloromycetin*" OR "vibramycin*" OR "levofloxacin*" OR "lymecycline*" OR "terramycin*" OR "spiramycin*"
#3	#1 OR #2
**#4 All Fields**	"Pregnancy" OR "Pregnant" OR "Maternal-exposure" OR (("gestation*" OR "in-utero" OR "mother*" OR "maternal*" OR "antenatal*" OR "prenatal*") AND ("use" OR "expose*" OR "exposure*" OR "usage")) OR ("vertical" AND "Transmi*") OR "Maternal-Fetal-Exchange" OR "Maternal-Foetal-Exchange" OR "maternal-fetal-transmission" OR "maternal-foetal-transmission" OR "fetomaternal-transfusion" OR "feto-maternal-transfusion"
**#5 All Fields**	"newborn*" OR "new-born*" OR "baby" OR "babies" OR "neonat*" OR "neo-nat*" OR "infan*" OR "fetus*" OR "fetus*" OR "fetal" OR "foetal" OR "toddler*" OR "child*" OR "offspring*" OR "pre-schooler*" OR "preschooler*" OR "kindergarten" OR "boy" OR "boys" OR "girl" OR "girls" OR "adolescen*" OR "pediatric*" OR "paediatric*" OR "youth*" OR "teen" OR "teens" OR "teenage*"
**#6 All Fields**	"hear*" OR "deaf*" OR "audiometr*" OR "cochlear-implant*" OR "cochlea-prosthes*" OR "evoked-response"
**#7 All Fields**	NOTNLM OR publisher[sb] OR inprocess[sb] OR pubmednotmedline[sb] OR indatareview[sb] OR pubstatusaheadofprint
#8	#3 AND #4 AND #5 AND #6 AND #7

Table S5. Publication bias

		Coefficient (95%CI)	Std. err	p value
Egger's test	slope	1.276 (1.058 ,1.495)	0.106	0.000
	bias	0.043 (-0.010, 0.096)	0.026	0.105
Trim-fill analysis		Effect size (95%CI)		
	Observed	0.119 (-0.018, 0.256)		
	Observed + Imputed	0.034 (-0.150, 0.219)		

Table S6. Summary of the quality assessment for the included studies

The Joanna Briggs Institute Critical Appraisal Checklist [1] comprised of 8 items with a maximum score of 8 points indicating the lowest possible risk of bias. Studies were judged to be at low (≥7), moderate (5-6), or high (≤4) risk of bias.[2]

The Newcastle-Ottawa Scale [3] comprised of 9 items for case-control and cohort studies and 10 items for cross-sectional studies. Studies were judged to be at low (≥8 points), moderate (5 to 7 points), or high (≤4 points) risk of bias.

The Cochrane Risk of Bias tool [4] comprised of 22 items. Studies were judged to be at low (≥7 points), moderate (5 to 6 points), or high (≤4 points) risk of bias.

8 Low 7 moderate 3 high

CASE REPORTS: Joanna Briggs Institute Critical Appraisal Checklist for Case Reports 2017 (L:>=6, M:3-5, H:2)

**STUDY ID: ROBINSON ET AL., 1964**				
**Major Components**	**Response options**			
1. Were patient's demographic characteristics clearly described?	Yes	No	Unclear	Not applicable
2. Was the patient's history clearly described and presented as a time-line?	Yes	No	Unclear	Not applicable
3. Was the current clinical condition of the patient on presentation clearly described?	Yes	No	Unclear	Not applicable
4. Were diagnostic tests or assessment methods and the results clearly described?	Yes	No	Unclear	Not applicable
5. Was the intervention(s) or treatment procedure(s) clearly described?	Yes	No	Unclear	Not applicable
6. Was the post-intervention clinical condition clearly described?	Yes	No	Unclear	Not applicable
7. Were adverse events (harms) or unanticipated events identified and described?	Yes	No	Unclear	Not applicable
8. Does the case report provide takeaway lessons?	Yes	No	Unclear	Not applicable
Overall appraisal: Include □ Exclude □ Seek further info □				

1 Low

**STUDY ID: JONES ET AL., 1973**				
**Major Components**	**Response options**			
1. Were patient's demographic characteristics clearly described?	Yes	No	Unclear	Not applicable
2. Was the patient's history clearly described and presented as a timeline?	Yes	No	Unclear	Not applicable
3. Was the current clinical condition of the patient on presentation clearly described?	Yes	No	Unclear	Not applicable
4. Were diagnostic tests or assessment methods and the results clearly described?	Yes	No	Unclear	Not applicable
5. Was the intervention(s) or treatment procedure(s) clearly described?	Yes	No	Unclear	Not applicable
6. Was the post-intervention clinical condition clearly described?	Yes	No	Unclear	Not applicable
7. Were adverse events (harms) or unanticipated events identified and described?	Yes	No	Unclear	Not applicable
8. Does the case report provide takeaway lessons?	Yes	No	Unclear	Not applicable
Overall appraisal: Include □ Exclude □ Seek further info □				

1 Low

CASE-CONTROL RISK OF BIAS SCORES (L:>=7, M:5-6, H:<=4)

**Study ID**	**Selection (out of 4)**	**Comparability (out of 2)**	**Exposure (out of 3)**	**Total Score (out of 9)**
**Xiong et al. 2017**	4	0	3	7 Low

**Foch et al. 2018**	3	1	3	7 Low
**Castillo et al. 2019**	4	0	2	6 Moderate

2 low 1 moderate

COHORT RISK OF BIAS SCORES (L:>=8, M:5-7, H:<=4)

**Study ID**	**Selection (out of 4)**	**Comparability (out of 2)**	**Outcome (out of 3)**	**Total Score (out of 9)**
**Conway et al. 1965**	2	0	3	5 Moderate
**Donald et al. 1991**	2	0	3	5 Moderate
**Kirkwood et al. 2007**	3	1	2	6 Moderate
**Leung et al. 2016**	4	1	3	7 Moderate
**DiStadio et al. 2019**	4	0	3	7 Moderate
**Patatt et al. 2021**	4	1	3	8 Low
**Tsao et al 2023**	3	2	3	8 Low
**Tsao et al 2024**	3	2	3	8 Low

3 low, 5 moderate

CROSS-SECTIONAL STUDIES: Newcastle-Ottawa Scale (adapted) L:>=7, M:5-6, H:=4

**Study ID**	**Selection (out of 4)**	**Comparability (out of 2)**	**Outcome (out of 3)**	**Total Score (out of 10)**
**Ganga et al. 1991**	0	0	2	2 High
**Jafari et al. 2007**	2	0	2	4 high
**Saunders et al. 2007**	3	0	2	5 Moderate
**Saunders et al. 2009**	2	0	0	2 High

3 high,1 moderate

RANDOMISED CONTROLLED TRIAL: RoB Cochrane

**STUDY ID**	**Risk of Bias Assessment Score**				
	**Domain 1 (Randomisation)**	**Domain 2 (Intervention Deviation)**	**Domain 3 (Missing outcome data)**	**Domain 4 (Outcome Measurement)**	**Domain 5 (Result selection)**
Lyell et al. 2010	Low	Low	Low	Low	Some concerns
**Overall Score = Low risk of bias**					

1 Low

Table S7. Summary of the included studies

Author Year	Location	Study Design	Data Collection Years	Sample size	Gestational age	In-utero exposure to antibiotics	Antibiotic use indication	Ascertainment of exposure	Hearing Loss Diagnosis Method	Age of Diagnosis	Hearing loss definition	Main findings	Confounding Adjustment
**Studies reporting associations between in-utero exposure to antibiotics exposure and hearing loss in offsprings**													
Tsao et al. 2024	Taiwan	Case control	2002-2015	3596 hearing loss cases, 17980 controls, total 21576	< 37 weeks	Gentamicin and furosemide	N/A	3 nationwide databases with maternal and child health medical records	Diagnosis at ≥2 outpatient clinics or 1 hospital admission within 1 year	Mean ± SD All: 1.95 ± 2.33 years<br><br>Cases: 1.74 ± 2.12 years<br><br>Controls: 1.99 ± 2.37 years	According to ICD-9-CM 389, 794.15	Gentamicin and furosemide cOR = 1.28 (1.19–1.38)<br><br>aOR 0.97 (0.89–1.06)	Maternal hearing loss, chorioamnionitis, pregnancy-induced hypertension, chronic hypertension, type 1 diabetes, type 2 diabetes, gestational diabetes, antenatal steroid and magnesium sulfate, multiple gestation, small for gestational age, gestational age ≤32 weeks
Tsao et al. 2023	Taiwan	Case control	2002-2015	12873 hearing loss cases, 64365 controls, total 77238	Full term	Gentamicin and furosemide		3 nationwide databases with maternal	Diagnosis at ≥2 outpatient clinics or 1 hospital	Mean± SD All: 2.79 ± 2.62 years	According to ICD-9-CM 389, 794.15	Gentamicin and furosemide<br><br>cOR = 1.18 (1.13–1.24)	Maternal hearing loss pregnancy-induced

								and child health medical records	admission within 1 year	Cases: 2.49 ± 2.43 years<br><br>Controls:2.85 ± 2.65 years		aOR 1.01 (0.96–1.07)	hypertension<br>chronic hypertension<br>type 1 diabetes<br>type 2 diabetes<br>gestational diabetes<br>maternal thyroid dysfunction<br>multiple births<br>small for gestational age
Castillo-Chávez et al. 2019	Mexico	Case control	2016-2017	22 cases, 105 controls, from one hospital	Gestational age range 30-37 weeks	Multiple: amikacin, ampicillin, cephalothin, cefalexin, cefotaxime, ceftriaxone, clindamycin, chloramphenicol, erythromycin, gentamicin<br><br>Dosage varies between	Unspecified	Medical records	TEOAE	0.5 years	Hearing threshold > 30 dB HL	Multiple antibiotics OR 0.72 (0.29, 1.81)	N/A

						antibiotics, in 3rd trimester							
Foch et al. 2018	France	Case control	1 July 2004 – 30 June 2015	1245 cases, 28046 controls	N/A	Gentamicin, erythromycin, tobramycin, netilmicin, unspecified trimester and dosage	Unspecified	Births and pregnancy database	Abnormal hearing on health certificate	2 years	N/A	Gentamicin OR = 3.22 (0.40, 26.19) Erythromycin OR = 1.11 (0.82, 1.50), Tobramycin OR = 0, Netilmicin OR = 0	N/A, but genetics excluded in sample selection,
Xiong et al. 2017	China	Case control	Mar 2014 - May 2015	118 cases, 242 controls, from 2 hospitals	N/A	Aminoglycosides, unspecified trimester and dosage	Maternal infections unspecified	Medical records and structured questionnaires	Congenital deafness, deafness or mutism at birth	Mean± SD Cases: 2.43 ± 1.05 years Controls: 2.57 ± 0.91 years	N/A	Aminoglycosides OR = 3.75 (1.08, 13.08)	N/A
Leung et al. 2016	United States	Retrospective cohort	Jun 1999- Jul 2003	289 babies in NICU, from 2 hospitals in Maryland	Gestational age < 33 weeks	Multiple: gentamicin, erythromycin, azithromycin, ampicillin/sulbactam, amoxcillin/clavulanate, metronidazole, clindamycin And others[%]	Group B Streptococcus Latency Suspected chorioamnionitis Maternal infection	Medical record	AABR	N/A	Hearing loss > 30 dB HL	ORs: Multiple abx 0.55 (0.28, 1.11), Others abx group 1.06 (0.51, 2.23), Gentamicin 1.82 (0.68, 4.85), Azithromycin 0.90 (0.11, 7.67), Ampicillin 0.64 (0.34, 1.21), Ampicillin/sulbactam 2.33 (1.09, 4.95),	N/A, specified adjustment only for ampicillin/sulbactam (gestational age)

						(Did not specify dosage or trimester of exposure)						Amoxicillin/clavulanate 0.67 (0.08, 5.49) Metronidazole 1.78 (0.62, 5.14) Clindamycin 1.00 (0.37, 2.76)	
**Studies reporting the number of hearing loss cases in children with mothers exposed to antibiotics in pregnancy**													
Patatt et al. 2021	Brazil	Cohort	Sept 2014 – Dec 2015	527 newborns from 2 sites in Porto Velho	Gestational age: 38.4-38.6 weeks	Clindamycin + Quinine, 3rd trimester		Interview and medical records	TEOAE and/or A-BAEP	N/A	N/A	No babies developed hearing loss	N/A
Stadio et al. 2019	Italy	Prospective cohort	Mar-Nov 2017	153 babies in NICU	Gestational age mean 33 weeks, range: 23-42 weeks	Unspecified antibiotics	Maternal infections#	Medical records,	TEOAE and AABR	N/A	Moderate (41-55 dB HL) Profound (>91 dB HL)	No babies developed hearing loss	N/A
Lyell et al. 2010	United States	Randomised controlled trial	Jun 2004 – Oct 2006	125 mothers from one California hospital	N/A	Group1: Daily Gentamicin* Group 2: 8-Hour Gentamicin** For Caesarean Birth: Clindamycin:	Intrapartum Chorioamnionitis	Clinical trial and exam	AABR	At birth	Failing hearing screen	No babies developed hearing loss	N/A

						900 mg every 8 hours (3 doses in total). Gestation:32-42 wks.							
Saunders et al. 2009	Nicaragua	Cross-sectional	N/A	31 cases, from an otolaryngology/audiology clinic	N/A	Gentamicin, unspecified timing and dosage	Meningitis Perinatal distress	Questionnaire	Air and bone conduction audiometry at 500 Hz, 1,000 Hz, 2,000 Hz, 4,000 Hz.	Before 18 years old	Mild (21-40dB) Moderate (41-60dB) Severe (61-80dB) Profound (> 80dB)	Only 2 cases with bilateral profound hearing loss were exposed to abx during pregnancy*	N/A
Saunders et al. 2007	Nicaragua	Cross-sectional	N/A	96 cases, from an otolaryngology/audiology clinic	N/A	Gentamicin, unspecified timing and dosage	Unspecified	Questionnaire	Air and bone conduction audiometry	During 1st 18 years of life	Mild (21-40dB) Moderate (41-60dB) Severe (61-80dB) Profound (> 80dB)	Only 2 cases with bilateral profound hearing loss were exposed to abx during pregnancy	N/A
Kirkwood et al. 2007	Canada	Cohort	Jan 2002 - Apr 2006	40 babies, from one hospital	N/A	Gentamicin, at least one dose, intravenous, mean dose ± SD: 764mg ± 600mg for	Maternal infections unspecified	Pharmacy records and chart review	TEOAE, and AABR	1st 3 days after birth	N/A	Out of 40 babies exposed, none developed hearing loss	Confounders identified but not adjusted for

						mean duration 2.7 ± 2.3 days, at average gestational age of 28 ± 6 weeks							
Jafari et al. 2007[7]	Iran	Cross-sectional	Jul-Dec 2005	86 children from Newsha Aural Rehabilitation Center (all children had hearing loss)	N/A	Gentamicin (1st trimester), Kanamycin (3rd trimester)	Unspecified	Questionnaire	Pure tone audiometry	Average age of diagnosis = 15.2 ± 9.3 months (range 1-36 months)	Bilateral profound hearing loss ≥ 91 dB HL	2 cases with mothers who took abx during pregnancy (1 gentamicin, 1 kanamycin)	N/A
Ganga et al. 1991	India	Cohort	N/A	78 students from a government school for deaf children	N/A	Streptomycin, unspecified trimester and dosage	Maternal infections: syphilis, chickenpox	Interview	Failing Rinne and Weber tests (air/bone conduction threshold)	N/A	N/A	2 out of the 78 students had been exposed to streptomycin	N/A
Donald et al. 1991	South Africa	Cohort	N/A, likely 1990-1991	30 children from Brooklyn hospital and nearby clinics	N/A	Streptomycin, duration ranged between 0.25-2 months, various trimesters throughout pregnancy	Tuberculosis	Hospital/clinic records	Failing unspecified hearing tests	13-48 months of age	N/A	Severe unilateral deafness in 1 child exposed during 1st trimester	Abnormalities (e.g. congenital hypotonia) were also present

Jones 1973	United States	Case Report	N/A	1 case	N/A	Kanamycin (1g IM per day, total dose 4.5g, at 28 weeks gestation	Urinary traction infections	Clinical records and examinations	Unspecified audiologic tests. No speech development, no response to sounds in the environment or outside field of vision	1 and 3 years of age	N/A	1 case passed hearing tests but did not react to sounds and difficulties with speech	N/A
Conway et al. 1965	England	Case series	1950-1959	17 children, from one London hospital	N/A	1g per day/3-days/bi-weekly/6-weekly/6-days streptomycin or myxtamycin (streptomycin + dihydrostreptomycin), total doses ranged 29-202g, varying trimesters of exposure	Tuberculosis	Case records	Air-conduction audiometry	Tests done on children during study, age range between 6-13 years	Mild > 20dB HL, Moderate > 40dB HL, Severe > 60dB HL	4 unilateral high-tone hearing loss cases (3 mild, 1 severe) had mothers who took abx 1g/day during pregnancy	N/A
Robinson et al. 1964	Canada	Case Report	N/A	2 cases	N/A	Case 1: 1g streptomycin biweekly between weeks	Tuberculosis	Case records	N/A	Case 1: diagnosed at ~4years	N/A	Case 1: severe bilateral SNHL,<br>Case 2: severe bilateral SNHL	N/A but unlikely for genetics to contribute to HL onset

						6-14 of pregnancy<br>Case 2: 1g streptomycin biweekly during last 4 months of pregnancy				Case 2: diagnosed at ~2-3 years old			from genetic tests

Note:

TEOAE, Transient Evoked Otoacoustic Emissions; A-BAEP, Automated Brain-stem Auditory Evoked Potential; AABR, Automated Auditory Brainstem Responses; SNHL: Sensorineural hearing loss

* Daily Gentamicin: Gentamicin: 5 mg/kg once daily. Placebo (Saline): Given at 8 and 16 hours. Ampicillin: 2g every 6 hours (4 doses in total).

**8-Hour Gentamicin: Gentamicin: 2 mg/kg loading dose, followed by 1.5 mg/kg at 8 and 16 hours. Ampicillin: 2g every 6 hours (4 doses in total)

.# Cytomegalovirus, Toxoplasma, Rubeola, Candida albicans, E.coli, Chlamydia, Syphilis, Mycoplasma and Hepatitis C virus.

% sulfamethoxazole and trimethoprim, cefotetan, ceftriaxone, cephalexin, cefazolin

Figures

(a) Aminoglycosides

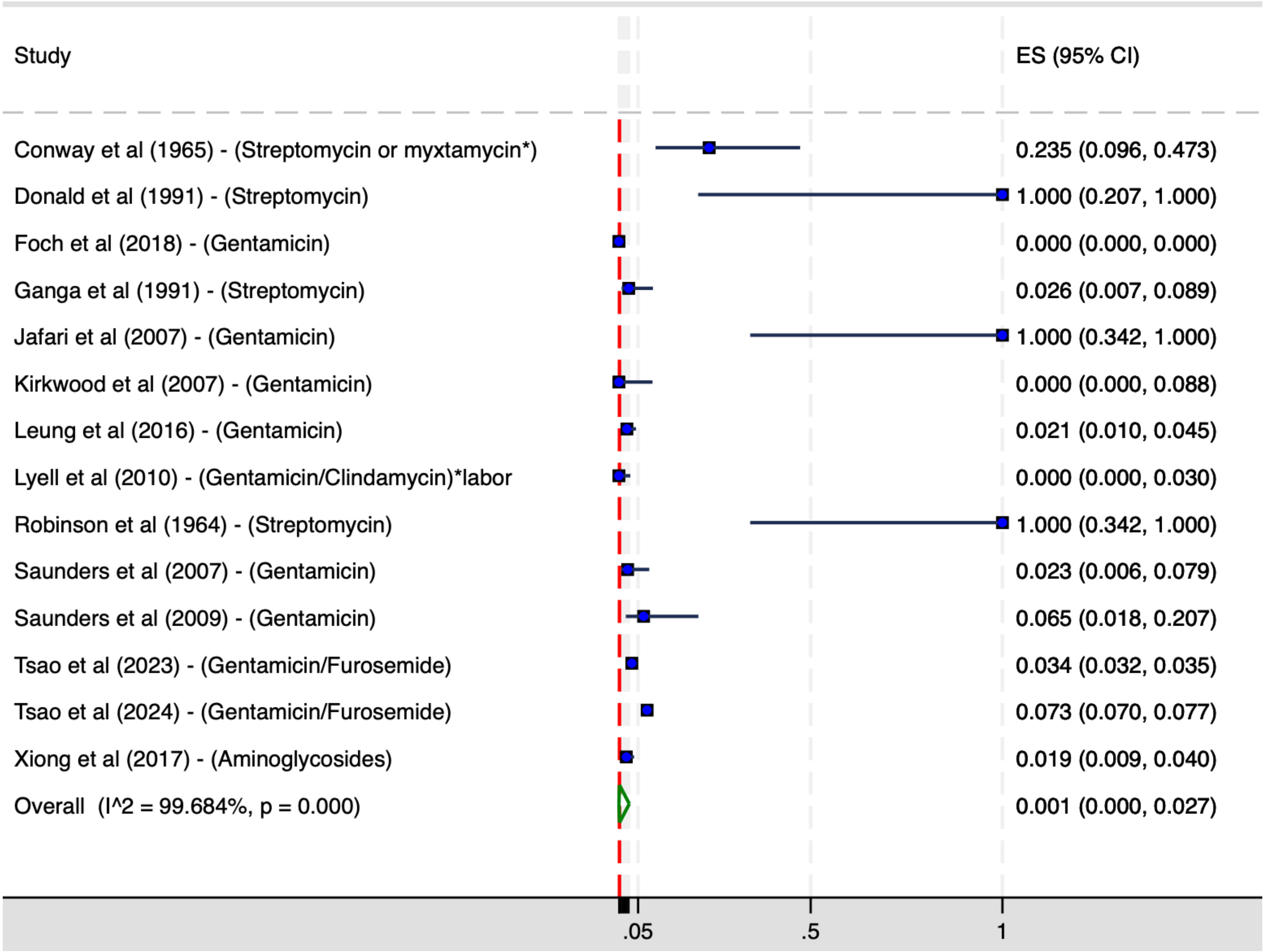

(b) Beta-Lactams

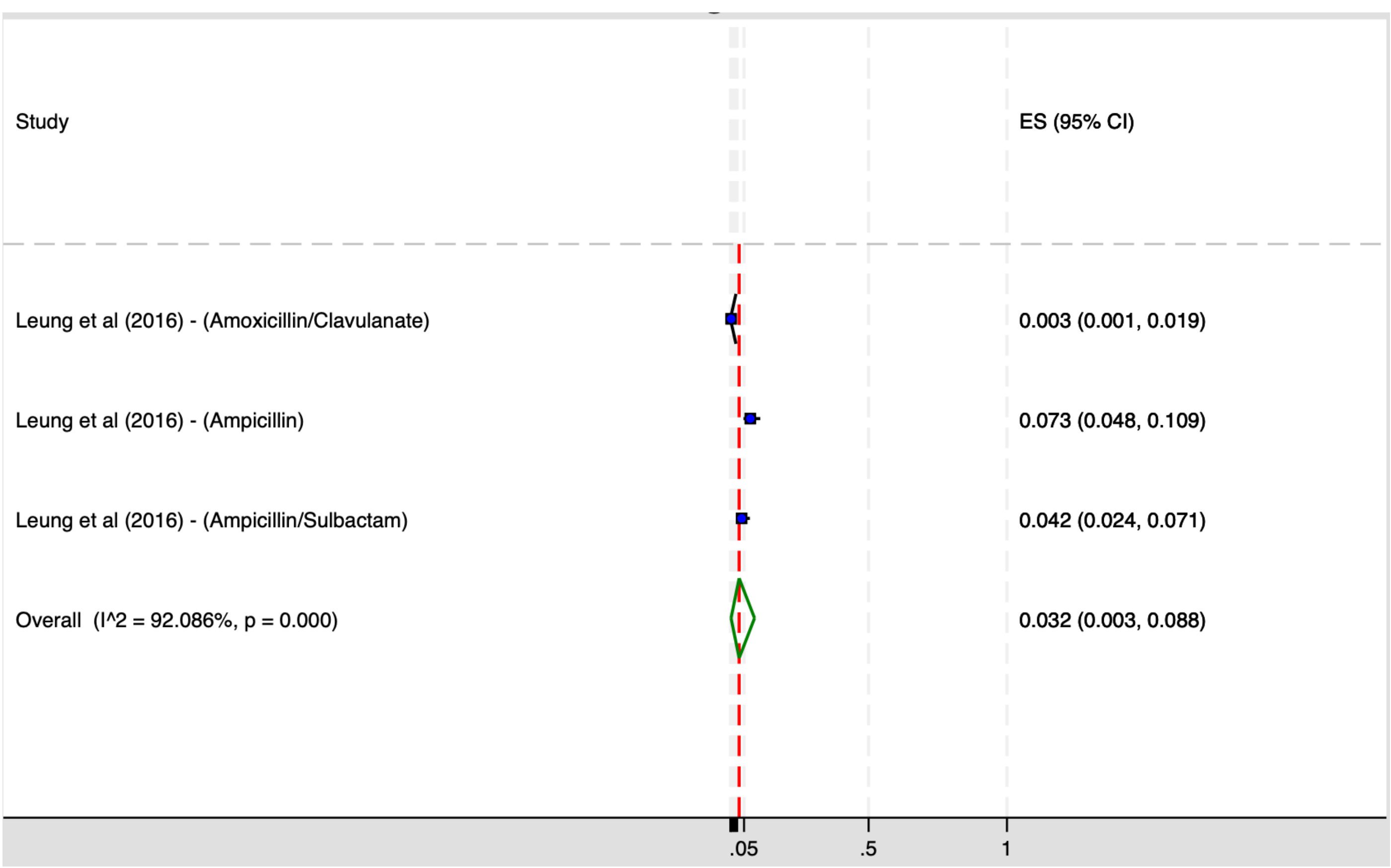

(c)Macrolides

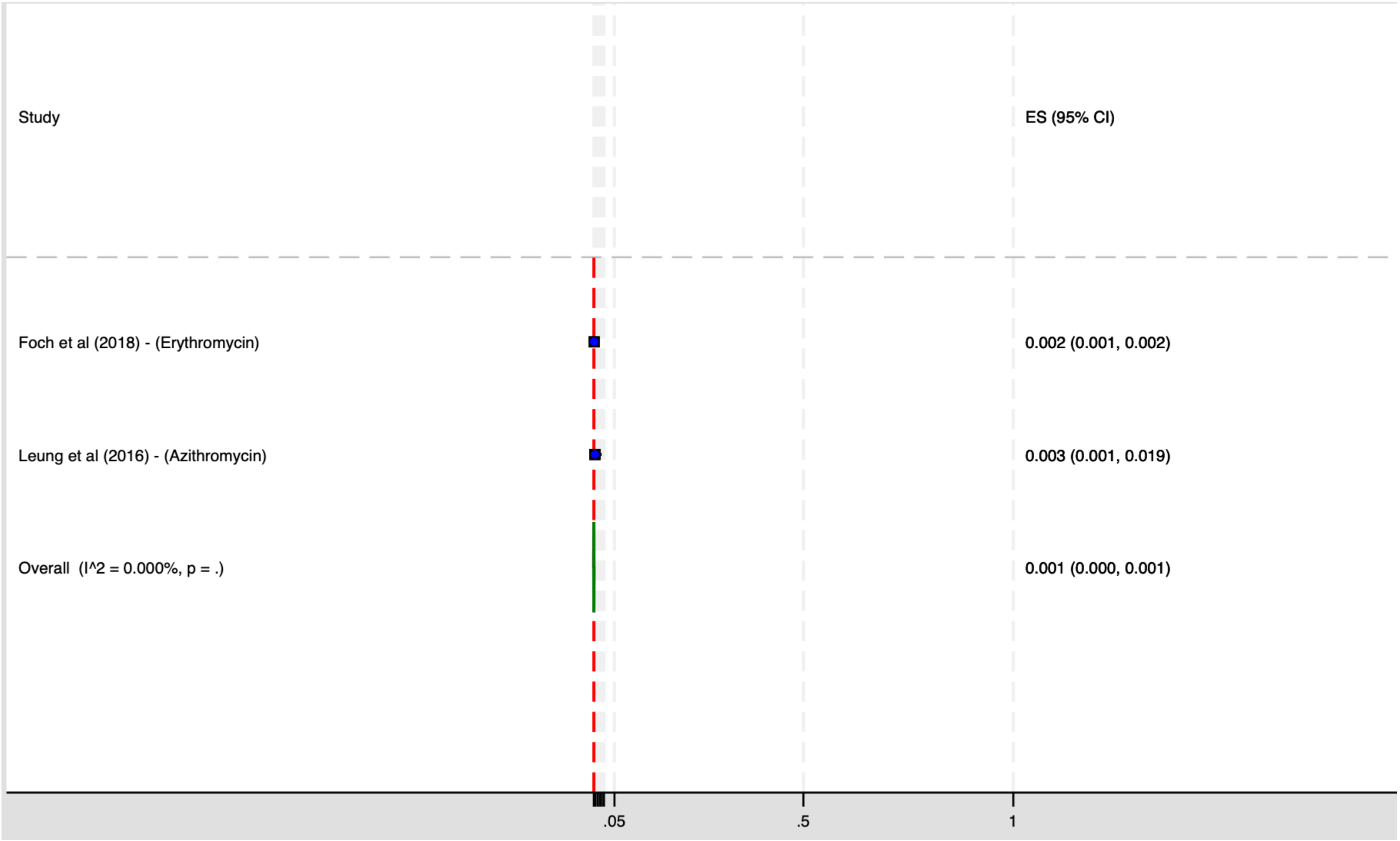

(d) Others (unspecified and Multiple classes)

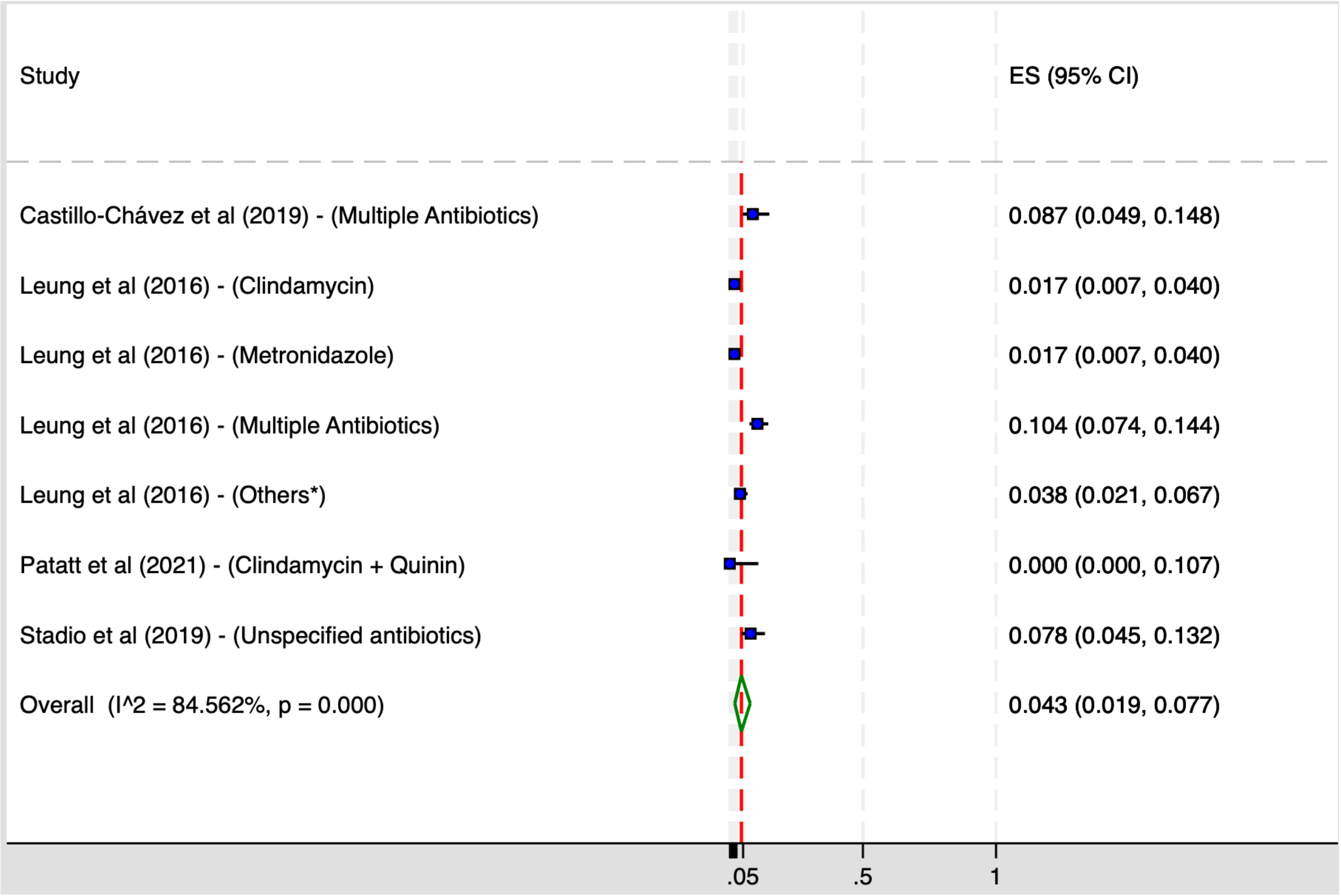

Figure S1. Pooled prevalence divided by different classes of antibiotics.

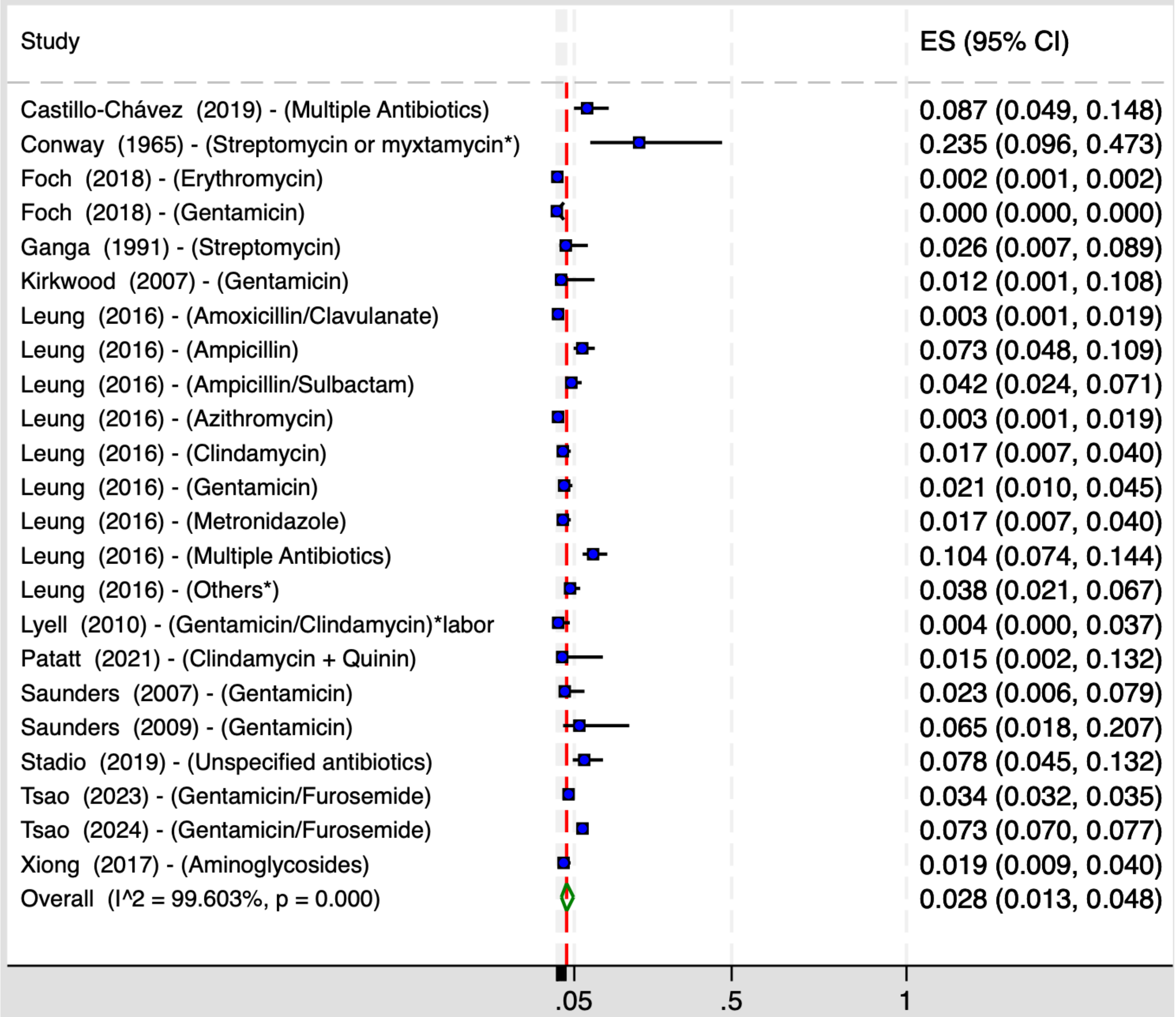

Figure S2. Sensitivity analysis on pooled prevalence

References

[1] Z. Munn, T. H. Barker, S. Moola, C. Tufanaru, C. Stern, A. McArthur, *et al.*, "Methodological quality of case series studies: an introduction to the JBI critical appraisal tool," *JBI Evid Synth,* vol. 18, pp. 2127-2133, Oct 2020.

[2] G. Rossi-Fedele, B. Kahler, and N. Venkateshbabu, "Limited Evidence Suggests Benefits of Single Visit Revascularization Endodontic Procedures - A Systematic Review," *Braz Dent J,* vol. 30, pp. 527-535, Nov-Dec 2019.

[3] G. A. Wells, B. Shea, D. O'Connell, J. Peterson, V. Welch, M. Losos, *et al.*, "The Newcastle-Ottawa Scale (NOS) for assessing the quality of nonrandomised studies in meta-analyses," 2000.

[4] J. A. C. Sterne, J. Savović, M. J. Page, R. G. Elbers, N. S. Blencowe, I. Boutron, *et al.*, "RoB 2: a revised tool for assessing risk of bias in randomised trials," *Bmj,* vol. 366, p. l4898, Aug 28 2019.

Disclaimer/Publisher's Note: The statements, opinions and data contained in all publications are solely those of the individual author(s) and contributor(s) and not of MDPI and/or the editor(s). MDPI and/or the editor(s) disclaim responsibility for any injury to people or property resulting from any ideas, methods, instructions or products referred to in the content.
